# Identification of shared genetic risks underlying metabolic syndrome and its related traits in the Korean population

**DOI:** 10.3389/fgene.2024.1417262

**Published:** 2024-07-10

**Authors:** Jun Young Kim, Yoon Shin Cho

**Affiliations:** Department of Biomedical Science, Hallym University, Chuncheon, Gangwon, Republic of Korea

**Keywords:** metabolic syndrome, genetic variation, genome-wide association study, genetic correlation, linkage disequilibrium score regression, Mendelian randomization

## Abstract

**Introduction:** Observational studies have demonstrated strong correlations between metabolic syndrome (MetS) and its related traits. To gain insight into the genetic architecture and molecular mechanism of MetS, we investigated the shared genetic basis of MetS and its related traits and further tested their causal relationships.

**Methods:** Using summary statistics from genome-wide association analyses of about 72,000 subjects from the Korean Genome and Epidemiological Study (KoGES), we conducted genome-wide multi-trait analyses to quantify the overall genetic correlation and Mendelian randomization analyses to infer the causal relationships between traits of interest.

**Results:** Genetic correlation analyses revealed a significant correlation of MetS with its related traits, such as obesity traits (body mass index and waist circumference), lipid traits (triglyceride and high-density lipoprotein cholesterol), glycemic traits (fasting plasma glucose and hemoglobin A1C), and blood pressure (systolic and diastolic). Mendelian randomization analyses further demonstrated that the MetS-related traits showing significant overall genetic correlation with MetS could be genetically determined risk factors for MetS.

**Discussion:** Our study suggests a shared genetic basis of MetS and its related traits and provides novel insights into the biological mechanisms underlying these complex traits. Our findings further inform public health interventions by supporting the important role of the management of metabolic risk factors such as obesity, unhealthy lipid profiles, diabetes, and high blood pressure in the prevention of MetS.

## Introduction

Metabolic syndrome (MetS) is a stopover for heart disease and many other chronic illnesses. It increases the probability of diabetes by about five times and that of cardiovascular disease by about two times, and individuals with MetS have a mortality rate four times higher than that of the general population. The prevalence of MetS is about 31.4% in the United States and about 20% in Korea, where it is increasing (Lee and Choi, 2022). In addition, MetS is strongly associated with fatty liver, obstructive sleep apnea, and various cancers. For example, MetS and its components are associated with the severity of acute pancreatitis (Niknam et al., 2020). Studies have also shown an association between MetS and other diseases such as COVID-19 (Jeon et al., 2022), cardiovascular disease (Ju et al., 2017), and pancreatic cancer (Zhong et al., 2023).

Both environmental and genetic factors are involved in MetS development. Although several environmental factors, including smoking, overeating, excessive drinking, abdominal obesity, and stress, contribute to the development of MetS, its pathogenesis has not been clarified. In this regard, numerous genetic association studies have been conducted to identify the genetic basis of MetS as a way to elucidate the molecular biological background of disease (Lind, 2019; Oh et al., 2020). Because MetS is a combination of metabolic risk factors, including obesity, dyslipidemia, insulin resistance, and elevated blood pressure (Kong and Cho, 2019), there have been efforts to identify the genetic factors for MetS-related diseases and quantitative traits (QTs) in addition to genetic studies of MetS (Wan et al., 2021). Furthermore, MetS-related traits may share genetic etiologies with each other.

To explore the extent to which genetic bases are shared across different traits, the method of linkage disequilibrium score regression (LDSC) has recently being applied in statistical genetics (Ni et al., 2018; van Rheenen et al., 2019). The genetic correlation between traits computed by LDSC refers to the correlation between the genetic effect on one trait and the genetic effect on another trait and can be used to explore novel trait associations. In this regard, it is possible to discover genes showing pleiotropy by identifying genetic correlations between clinically or physiologically related traits (van Rheenen et al., 2019).

Mendelian randomization (MR) is a method that uses genetic variations to determine whether the observed association of one trait as a risk factor with another trait as an outcome is consistent with a causal relationship (Zheng et al., 2019). MR relies on a natural and random classification of genetic variants during meiosis to produce a random distribution of genetic variation in a population. Because this approach takes advantage of the fact that the genotype precedes the living environment (McArdle et al., 2012), MR has been used to evaluate causal relationships between two traits.

In this study, we aimed to unravel the genetic correlation between MetS and its related traits and ultimately elucidate the genetic architecture underlying the development of MetS. In addition, we used MR to examine the causal relationships between MetS and its related traits. The outcomes of these approaches are expected to be valuable for MetS management and control in the human population.

## Materials and methods

### Study subjects

Subjects for genome-wide association (GWA) analyses of MetS and its related traits were recruited from the Korean Genome and Epidemiological Study (KoGES) that was established to investigate the genetic and environmental factors as determinants of the incidence of chronic diseases [such as type 2 diabetes (T2D), hypertension, obesity, metabolic syndrome, osteoporosis, cardiovascular disease, and cancer] by the Korean government (National Research Institute of Health, Korea Disease Control and Prevention Agency) since 2001 (Kim et al., 2016). In the present study, we used epidemiological data from about 72,000 individuals from three population-based cohorts of the KoGES, namely, the Korea Association Resource Study (KARE) cohort (Cho et al., 2009), the Health EXAminee shared control study (HEXA) cohort (Kim et al., 2011; Kim and Cho, 2023), and the CArdioVascular disease Association Study (CAVAS) cohort (formerly Health2 or RURAL cohort) (Cho et al., 2009). In brief, KARE cohort consists of two population-based studies, the rural Ansung and urban Ansan cohort studies, which were designed to allow longitudinal prospective study. Since the baseline study (including 10,038 participants aged 40–69) in 2001, the 11th follow-up study was scheduled for completion in late 2024. HEXA cohort was initiated in 2004 to identify environmental and genetic risk factors for major chronic diseases in Koreans, targeting men and women over 40 years of age who visited medical examination centers in urban areas. Initiated in 2005, CAVAS cohort was designed to investigate the effects of lifestyle habits, diet, and environmental factors on chronic disease development for rural residents. Signed informed consent was voluntarily received from all KoGES participants before the study. The study protocol was approved by the Institutional Review Boards of the institutions participating in KoGES.

### Genotyping, quality control, and imputation

This study used genotype data publicly available from the National Biobank of Korea (NBK), Korea National Institute of Health (https://biobank.nih.go.kr/cmm/main/mainPage.do). Genotyping of about 72,000 subjects from the three population-based cohorts of the KoGES was conducted using the Korea Biobank Array (KBA) chip (Moon et al., 2019). As genotype data quality control, samples with call rates <97%, excessive heterozygosity (HET) based on all variants on the array (HET <0.15 or HET >0.19), high singletons, gender mismatch, and second-degree relatives were removed. KING v2 was used to inferring 2nd-degree relatives (Manichaikul et al., 2010). Genetic variants [mostly single nucleotide polymorphisms (SNPs)] with a call rate <95%, minor allele frequency (MAF) <0.01, and Hardy-Weinberg equilibrium (HWE) *p* < 1 × 10^−6^ were excluded from subsequent association analyses (Kim and Cho, 2023). To extend SNP coverage, SNP imputation was performed using the IMPUTE4 program for phased genotype data with Eagle v2.3 software. The 1,000 Genomes Project Phase 3 and the Korean reference genome were used as a reference panel for SNP imputation. After imputation, SNPs with an INFO score <0.8 and MAF <0.01 were removed.

### Phenotyping

MetS cases were diagnosed according to International Diabetes Foundation (IDF) criteria (Yoon et al., 2007; Huang, 2009). MetS criteria include any three of the following factors: 1) systolic blood pressure (SBP) ≥130 mmHg, diastolic blood pressure (DBP) ≥85 mmHg, or taking blood pressure medication, 2) fasting plasma glucose (FPG) ≥100 mg/dL or taking diabetes medication, 3) triglycerides (TG) ≥150 mg/dL or taking lipid-lowering medication, 4) high-density lipoprotein cholesterol (HDLC) ≤40 mg/dL in men or <50 mg/dL in women or taking lipid-lowering medication, and 5) a waist circumference (WC) ≥90 cm in men or ≥85 cm in women. The MetS controls comprised individuals with traits not falling into any of the MetS factors (Table 1).

**TABLE 1:** Clinical statistics of subjects with MetS in the KBA dataset.

Trait	Variable	Case	Control
MetS	N	11,139	45,020
	Age (year)	54.85	52.29
	BMI (kg/m2)	25.87	23.18
	WC (cm)	87.25	78.50
	TG (mg/dL)	204.91	105.79
	HDLC (mg/dL)	42.91	55.21
	LDLC (mg/dL)	117.10	120.01
	TCHL (mg/dL)	204.96	196.92
	FPG (mg/dL)	99.35	88.21
	HbA1C (%)	2.97	2.98
	SBP (mmHg)	131.06	118.10
	DBP (mmHg)	82.02	74.01
Obesity	N	17,023	39,429
T2D	N	3,778	33,416
Dyslipidemia	N	19,951	37,582
Hypertension	N	17,429	48,372

Abbreviations are as follows: N, number of subjects; BMI, body mass index; WC, waist circumference; TG, triglyceride; HDLC, high-density lipoprotein cholesterol; LDLC, low-density lipoprotein cholesterol; TCHL, total cholesterol; FPG, fast plasma glucose; HbA1C, hemoglobin A1c; SBP, systolic blood pressure; DBP, diastolic blood pressure.

MetS component diseases such as obesity, dyslipidemia, T2D, and hypertension were assessed based on the diagnostic criteria of each disease (Inzucchi, 2012; Jordan et al., 2018; Halawani et al., 2019; Ahn et al., 2020) (Supplementary Table S1). In detail, the obesity cases were grouped for subjects with body mass index (BMI) >25.0 kg/m^2^, while controls were grouped for those with BMI between 18.5 and 22.9 kg/m^2^.

The T2D cases were diagnosed according to the following criteria: 1) treatment of T2D, 2) fasting plasma glucose ≥7 mmol/L or plasma glucose 2-h after ingestion of 75 gm oral glucose load ≥11.1 mmol/L. The inclusion criteria of nondiabetic control subjects were as follows: 1) no history of diabetes and 2) fasting plasma glucose <5.6 mmol/L and plasma glucose 2-h after ingestion of 75 gm oral glucose load <7.8 mmol/L.

The dyslipidemia cases were diagnosed if subjects had total cholesterol (TCHL) ≥240 mg/dL, low-density lipoprotein cholesterol (LDLC) ≥160 mg/dL, HDLC <40 mg/dL, TG ≥200 mg/dL, recent records of lipid-lowering medication, or dyslipidemia history. Subjects who did not meet all of these conditions were grouped as controls.

The hypertension cases were diagnosed for subjects with SBP ≥140 mmHg or DBP ≥90 mmHg. On the other hand, the controls for hypertension were grouped if subjects had SBP ≥120 mmHg and DBP ≥80 mmHg.

The demographic and clinical data of MetS-related traits such as obesity traits [body mass index (BMI) and WC], lipid traits [TG, HDLC, low-density lipoprotein cholesterol (LDLC), and total cholesterol (TCHL)], glycemic traits [FPG and hemoglobin A1C (HbA1C)], and blood pressure (SBP and DBP) were obtained from the KARE, HEXA, and CAVAS cohorts (Table 1; Supplementary Table S1).

### Statistical analyses

GWA analyses were conducted using the KBA dataset of KoGES subjects to identify genetic variants influencing MetS and its related traits. For MetS, logistic regression analysis was performed with adjustment for age, sex, and recruitment area. For MetS-related traits, linear regression analysis was performed with the above-mentioned adjustments. All association analyses were carried out via an additive model using PLINK v1.07 (https://zzz.bwh.harvard.edu/plink/) (Purcell et al., 2007).

A genetic correlation score (*r*_*g*_) was calculated using LDSC software (https://github.com/bulik/ldsc) to detect genetic correlations between MetS and its related traits (Bulik-Sullivan et al., 2015). Summary statistics from GWA analyses were used in the *r*_*g*_ calculation for the pairwise traits of interest.

MR analyses were carried out to test causal relationships between MetS and its related traits. Lead SNPs from GWA analyses of traits considered risk factors were selected as instrumental variables (IVs) to detect a causal relationship with the other trait considered an outcome. In the examples of this study, the odds of MetS risk were divided by the β coefficients of the levels of MetS-related traits to determine ratio estimates for each IV. The effects of the individual IVs were combined using inverse-variance weighted (IVW) analysis (Mounier and Kutalik, 2023), resulting in a weighted mean estimate of the risk of MetS per 1-standard deviation increase in the levels of MetS-related traits (diseases or QTs). In this study, MR analyses were conducted using the MendelianRandomization package in R software (version 4.3.0).

## Results

### Identification of genetic variants associated with MetS and related traits

Prior to genetic correlation and MR analyses, we conducted GWA analyses of MetS and its related traits. Subjects taking lipid-lowering, hypertension, or diabetes medication were excluded from the GWA analyses to minimize the confounding effects of the medication on the traits of interest in association analyses. The number of subjects in the analyses is summarized in Table 1; Supplementary Table S1.

**FIGURE 1:**
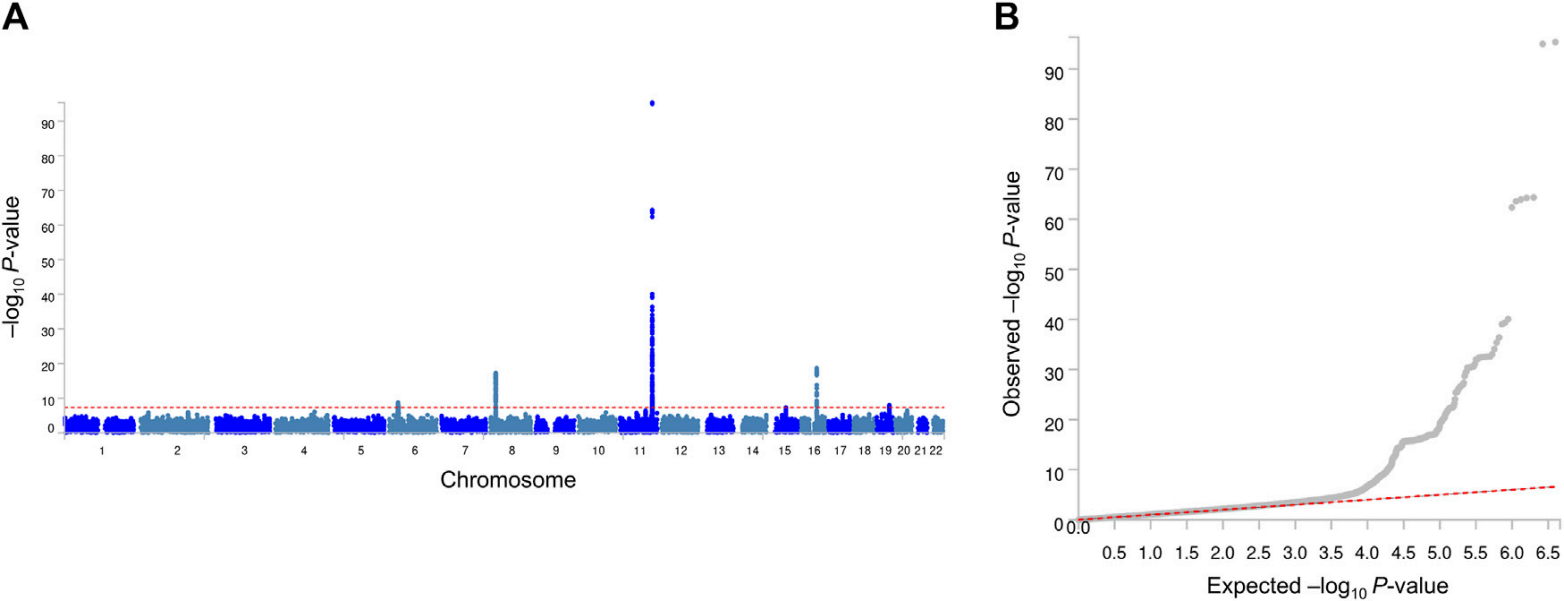
Manhattan plot **(A)** and quantile–quantile plot **(B)** of GWA analyses of MetS. In the Manhattan plot, the negative logarithm of the association *P*-value for each SNP across the whole genome is represented by a dot. The red line indicates the genome-wide significant *P*-value (5.0 × 10^−8^). In the quantile–quantile plot, the *x*- and *y*-axes represent the expected and observed *P*-values, respectively.

Our GWA analyses revealed significant associations for MetS (Figure 1) and its related traits (Supplementary Tables S2–S12; Supplementary Figures S1, S2). Because observational studies have reported strong correlations between MetS and its related traits, we inspected whether MetS loci also show an association with the related traits. In this study, four genome-wide significant (association *P*-value <5 × 10^−8^) MetS loci in *APOA5* (rs651821), *CETP* (rs56156922), *LPL* (rs4244457), and *APOE* (rs429358) also showed strong evidence of an association with lipid traits, implying a genome-wide genetic correlation between MetS and lipid traits (Table 2).

**TABLE 2:** Association of MetS SNPs with its related traits.

SNP		rs651821	rs56156922	rs4244457	rs429358
BP		11:116662579	16:56993886	8:19852156	19:45411941
Candidate gene		APOA5	CETP	LPL	APOE
Mi/Ma		C/T	G/A	T/G	C/T
MAF		0.29	0.18	0.34	0.09
MetS	OR	1.40	0.83	0.87	1.15
	*P*-value	4.27 × 10^−96^	2.20 × 10^−19^	5.81 × 10^−18^	1.09 × 10^−9^
BMI	β	−0.02	0.02	0.00	−0.03
	*P*-value	3.57 × 10^−1^	2.16 × 10^−1^	9.74 × 10^−1^	3.22 × 10^−1^
WC	β	−0.01	0.01	−0.02	−0.15
	*P*-value	9.01 × 10^−1^	8.68 × 10^−1^	7.50 × 10^−1^	3.68 × 10^−2^
TG	β	25.88	−3.02	−13.43	11.61
	*P*-value	<0.01	7.33 × 10^−7^	4.02 × 10^−84^	3.88 × 10^−50^
HDLC	β	−2.51	3.81	2.18	−1.72
	*P*-value	2.04 × 10^−271^	<0.01	6.06 × 10^−108^	9.09 × 10^−54^
LDLC	β	−0.39	0.23	1.32	4.56
	*P*-value	3.68 × 10^−2^	3.02 × 10^−1^	1.96 × 10^−7^	2.86 × 10^−56^
TCHL	β	1.24	3.62	1.17	4.45
	*P*-value	9.82 × 10^−10^	1.30 × 10^−48^	3.24 × 10^−5^	6.07 × 10^−45^
FPG	β	0.20	0.00	−0.32	0.05
	*P*-value	7.28 × 10^−2^	9.97 × 10^−1^	4.38 × 10^−2^	7.71 × 10^−1^
HbA1C	β	0.00	−0.01	−0.01	−0.01
	*P*-value	4.21 × 10^−1^	6.16 × 10^−2^	1.54 × 10^−1^	5.28 × 10^−1^
SBP	β	0.13	0.02	0.01	−0.43
	*P*-value	1.14 × 10^−1^	8.60 × 10^−1^	9.42 × 10^−1^	1.36 × 10^−3^
DBP	β	0.11	0.03	−0.02	−0.29
	*P*-value	4.19 × 10^−2^	6.58 × 10^−1^	7.97 × 10^−1^	8.55 × 10^−4^

Information for the SNP ID and chromosomal position is based on NCBI genome build 37/hg19. Abbreviations are as follows: BP, base-pair (Physical position); Mi/Ma, minor allele/major allele; MAF, minor allele frequency; MetS, metabolic syndrome; BMI, body mass index; WC, waist circumference; TG, triglyceride; HDLC, high-density lipoprotein cholesterol; LDLC, low-density lipoprotein cholesterol; TCHL, total cholesterol; FPG, fast plasma glucose; HbA1C, hemoglobin A1c; SBP, systolic blood pressure; DBP, diastolic blood pressure.

### Quantification of the genome-wide genetic correlation between MetS and related traits

**FIGURE 2:**
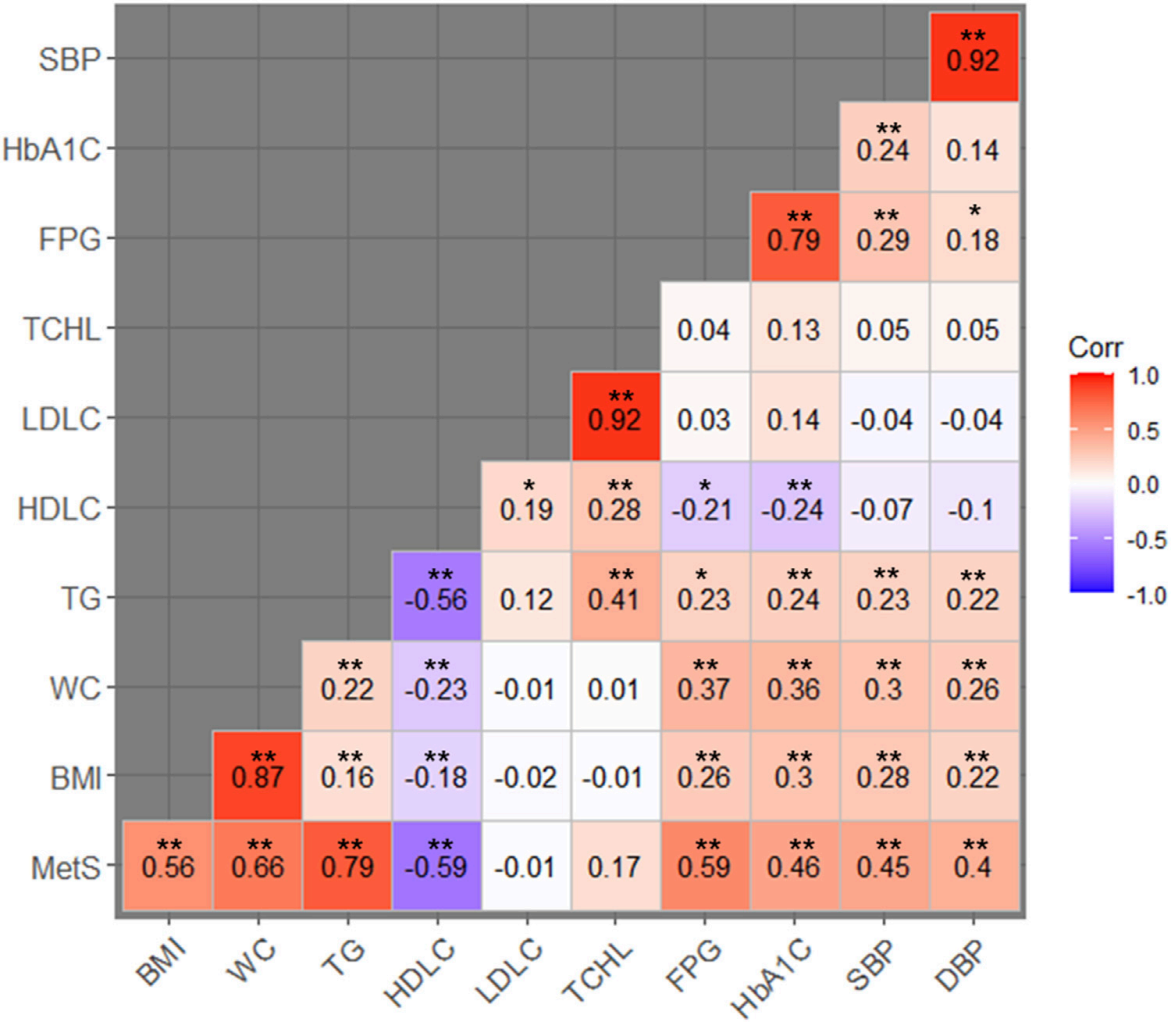
Heat map of the genetic correlation between MetS and its related traits. Red-colored boxes represent positive genetic correlations while blue-colored boxes represent negative correlations. Numbers in the boxes indicate the genetic correlation coefficients between the traits being compared. *Genetic correlations with *P*-values less than 0.05; **genetic correlations with *P*-values less than 0.01.

Based on the evidence from the cross-trait associations of single markers (Table 2), we attempted LD score regression analysis to estimate the correlation of phenotypic effects of genetic variants across the genome on two traits (in this case, MetS and one of the related traits). We estimated the genome-wide genetic correlation between MetS and its related traits (e.g., BMI, WC, TG, HDLC, LDLC, TCHL, HbA1C, SBP, and DBP). We observed a significant genetic correlation between MetS and most of the related traits, except LDLC and TCHL (Table 3; Figure 2). Of these, TG showed the strongest positive genetic correlation with MetS (*r*_*g*_ = 0.79, *P*-value = 1.19 × 10^−46^). On the other hand, HDLC showed a negative genetic correlation with MetS (*r*_*g*_ = −0.59, *P*-value = 2.74 × 10^−10^). These results are in good agreement with observations from physiological, clinical, and epidemiological studies.

**TABLE 3:** Genome-wide genetic correlations between MetS and its related traits.

Trait 1	Trait 2	*r*_*g*_	*r*_*g*_ SE	*P*-value
MetS	BMI	0.56	0.063	3.12 × 10^−19^
	WC	0.66	0.063	4.25 × 10^−26^
	TG	0.79	0.055	1.19 × 10^−46^
	HDLC	−0.59	0.093	2.74 × 10^−10^
	LDLC	−0.01	0.098	9.40 × 10^−1^
	TCHL	0.17	0.100	8.69 × 10^−2^
	FPG	0.59	0.085	4.18 × 10^−12^
	HbA1C	0.46	0.097	2.34 × 10^−6^
	SBP	0.45	0.074	1.26 × 10^−9^
	DBP	0.40	0.074	7.73 × 10^−8^

Abbreviations are as follows: QT, quantitative trait; r_g_, genetic correlation; SE, standard error; MetS, metabolic syndrome; BMI, body mass index; WC, waist circumference; TG, triglyceride; HDLC, high-density lipoprotein cholesterol; LDLC, low-density lipoprotein cholesterol; TCHL, total cholesterol; FPG, fast plasma glucose; HbA1C, hemoglobin A1c; SBP, systolic blood pressure; DBP, diastolic blood pressure.

### Inference of a causal relationship between MetS and related traits

**FIGURE 3:**
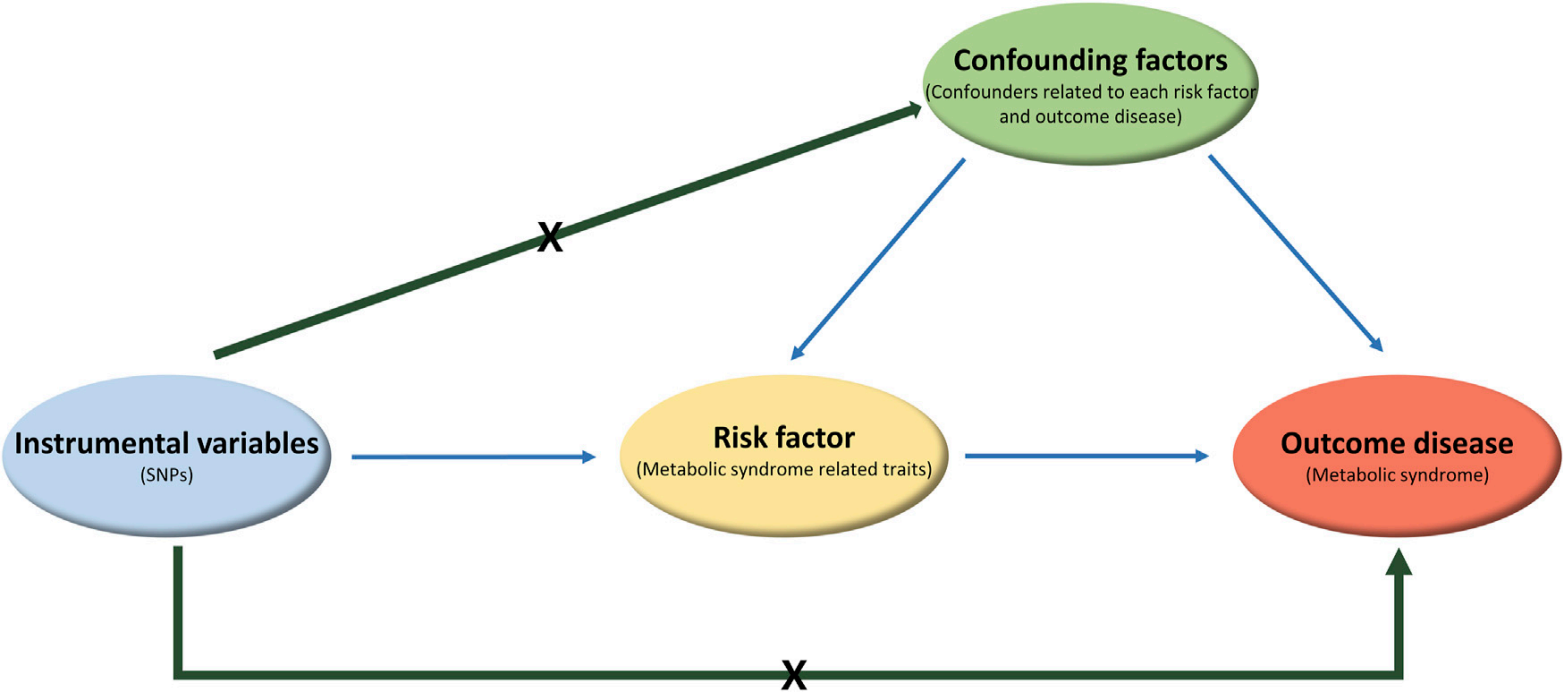
Diagram displaying the components of the Mendelian randomization. Genetic variants as the instrumental variables are associated with risk factor (or exposure), but not with confounding factors or outcome disease. Biomarker is a modifiable risk factor for outcome disease.

**FIGURE 4:**
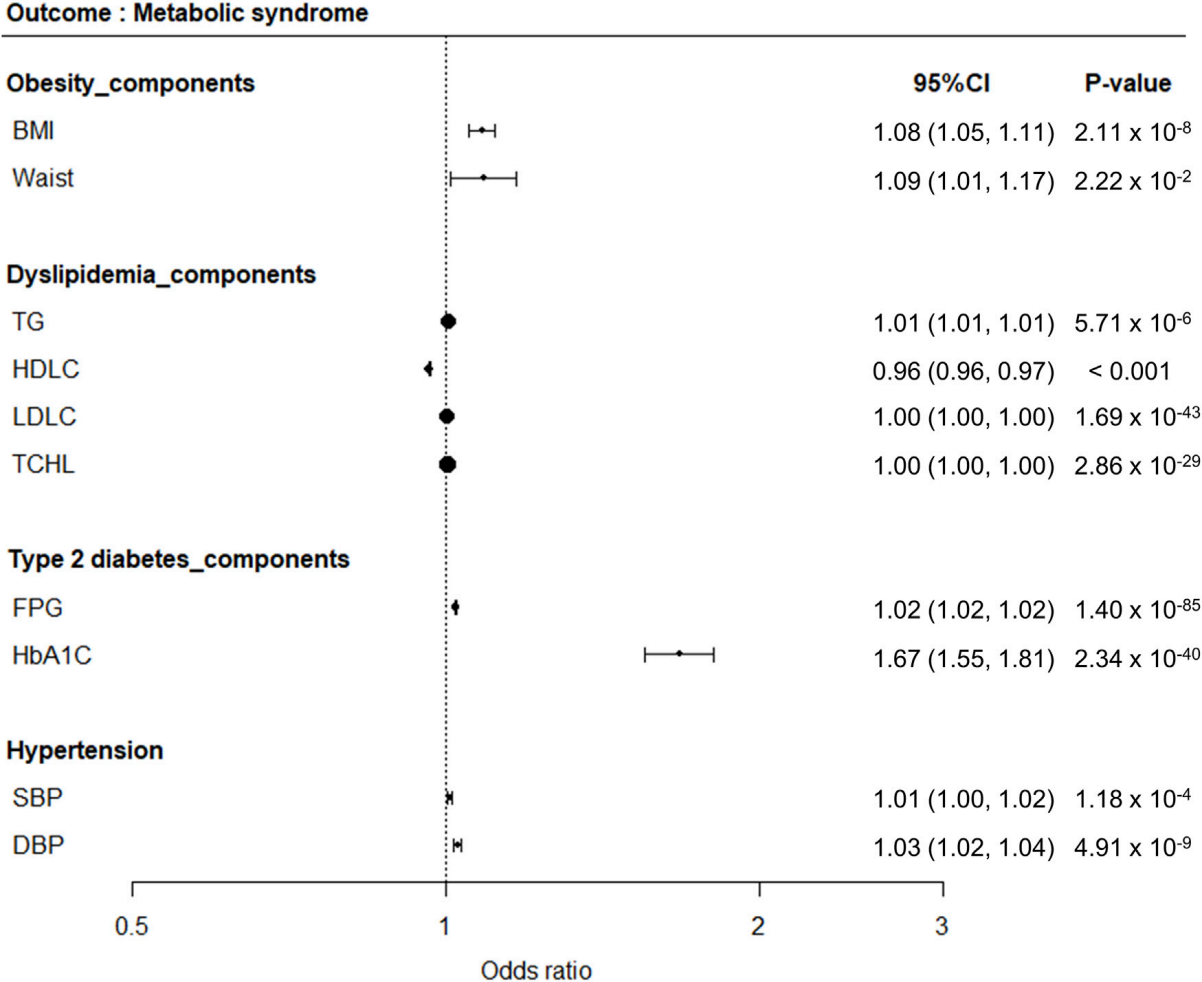
Forest plots demonstrating results of MR analyses between MetS and its related traits. MR analyses were conducted to detect causal relationships between MetS as an outcome disease and its related traits as risk factors.

One-sample MR analysis applying an IVW approach was performed to assess the causal relationship between MetS and its related traits (Figure 3). We detected a significant causal relationship between MetS and its related traits, such as BMI, WC, TG, HDLC, FPG, HbA1C, SBP, and DBP (Figure 4). The genetically determined risk factors of HDLC showed a protective effect on MetS risk [odds ratio (OR): 0.96, *P* < 0.001], whereas BMI (OR: 1.08, *P* = 2.11 × 10^−8^), WC (OR: 1.09, *P* = 2.22 × 10^−2^), TG (OR: 1.01, *P* = 5.71 × 10^−6^), FPG (OR: 1.02, *P* = 1.40 × 10^−85^), HbA1C (OR: 1.67, *P* = 2.34 × 10^−40^), SBP (OR: 1.01, *P* = 1.18 × 10^−4^), and DBP (OR: 1.03, *P* = 4.91 × 10^−9^) were significantly associated with an increased risk of MetS.

## Discussion

Based on observational studies showing strong correlation between MetS and its related traits, we investigated shared genetic basis underlying these traits to gain insight into the genetic architecture and molecular mechanism of MetS. Our LDSC analyses demonstrated a significant genetic correlation of MetS with obesity traits (BMI and WC), lipid traits (TG and HDLC), glycemic traits (FPG and HbA1C), and blood pressure (SBP and DBP). MR analyses further demonstrated that the MetS-related traits showing significant overall genetic correlation with MetS could be genetically determined risk factors for MetS.

Numerous genetic association studies have been conducted to understand the genetic basis of MetS as a primary way to elucidate the molecular biological background of disease. More than 100 independent loci for MetS were thus far identified from GWASs in diverse ethnic populations (Lind, 2019; Oh et al., 2020). In our GWA analysis of MetS, we identified several genome-wide significant loci located in or near genes such as *APOA5*, *CETP*, *LPL*, and *APOE*, which are mostly involved in lipid metabolism. In addition, our study also detected several loci showing a suggestive association with MetS; these loci are involved in diabetes, obesity, and hypertension.

The protein encoded by *APOA5*, apolipoprotein A5 (APOA5), plays an important role in regulating plasma TG levels and as a major risk factor for coronary artery disease (Pennacchio et al., 2002). As a component of high-density lipoprotein, APOA5 is also associated with lipid-related diseases such as hypertriglyceridemia 1 and hyperlipoproteinemia type V (Johansen et al., 2010; Mendoza-Barbera et al., 2013). The *CETP*-encoded protein cholesteryl ester transfer protein (CETP) is involved in the transfer of cholesteryl ester from high-density lipoprotein to other lipoproteins (Inazu et al., 1990). Diseases such as hyperalphalipoproteinemia 1 and lipid metabolism disorder are associated with CETP (Nagano et al., 2002). The protein encoded by *LPL* is lipoprotein lipase (LPL), which, as a homodimer, catalyzes the hydrolysis of triglycerides from circulating chylomicrons and very-low-density lipoproteins (Emi et al., 1990). Thus, LPL plays an important role in lipid clearance from the blood stream and in lipid utilization and storage. Mutations in LPL are involved in type I hyperlipoproteinemia and many disorders related to lipoprotein metabolism (Wilson et al., 1993). The coding product of *APOE*, apolipoprotein E (APOE), associates with lipid particles, which mainly function in lipoprotein-mediated lipid transport between organs via the plasma and interstitial fluids. APOE, as a core component of plasma lipoproteins, is involved in their production, conversion, and clearance (Verghese et al., 2013) and is associated with lipoprotein glomerulopathy and hyperlipoproteinemia type III (Evans et al., 2005; Rovin et al., 2007).

In addition to our study, previous GWASs showed that a considerable number of MetS loci overlapped those discovered for two or more MetS-related traits (Kristiansson et al., 2012; Lee et al., 2018; Lind, 2020). Although these findings may mirror the results of observational clinical and epidemiological studies showing significant correlations between MetS and its related traits, including diseases and QTs, their genetic overlap is less well elucidated (Cherny et al., 2022).

Genetic correlations have been estimated between MetS traits in Europeans (Vattikuti et al., 2012; van Walree et al., 2022). In this study, we used LDSC analysis to examine the cross-trait genetic correlations in Korean populations to gain insight into the shared genetic basis of MetS and its related traits in East Asians. To the best of our knowledge, our study is the largest genome-wide cross-trait genetic correlation analysis of MetS and its related traits in East Asians. Another advantage of our study is that we ruled out confounding effects as much as possible by eliminating subjects who took lipid-lowering, hypertension, or diabetes medication from the GWA analyses of MetS and its related traits. Finally, our results demonstrated a positive overall genetic correlation of MetS with obesity, glycemic, and blood pressure traits but a negative correlation with HDLC. These findings are largely consistent with those of conventional epidemiological studies.

In addition to comparing MetS and its related traits, we investigated genetic correlations among MetS-related traits. In our analyses, most MetS-related traits, including obesity traits (BMI and WC), glycemic traits (FPG and HbA1C), lipid traits (TG, HDLC, LDLC, and TCHL), and blood pressure (SBP and DBP), showed significant genetic correlations when analyzed in pairs, except LDLC and TCHL. These results are largely consistent with those observed in Europeans where pairwise analyses were performed for WC, FPG, TG, HDLC, and SBP (van Walree et al., 2022).

Findings on the overall genetic correlation between MetS and its related traits suggest a shared genetic basis for pairs of traits, which is either directly through variants affecting both traits (pleiotropy) or through the causal effect of one trait on the other. In this regard, we further exploited the causal relationships between MetS and its related traits by applying MR. In our one-sample MR analysis, the outcome was MetS and the risk factor was one of the MetS-related traits. According to the key assumptions of MR, the genetic variant, as the instrumental variable (IV) that is causally related to the risk factor, should only affect the outcome through its effect on the risk factor. In addition, confounding factors for the association between risk factor and outcome should not be related to the IV.

To meet the above-stated assumptions of MR, we performed MR analysis using variants associated with risk factors after excluding variants showing an association with confounding factors. Our MR analyses demonstrated the causal relationships of MetS-related traits (e.g., BMI, WC, TG, HDLC, FPG, HbA1C, SBP, and DBP) with MetS. To the best of our knowledge, our MR results are the first to show a causal relationship between MetS and its related traits, building on the results of genetic correlation analyses. In conclusion, the results of our MR analyses highlighted the causal role of MetS-related traits in MetS development. These findings suggest that treatment of each of the MetS-related traits individually may be a valuable strategy in the clinical management of MetS. However, this study was mainly limited to the Korean population. Therefore, our findings should be further validated in different ancestral populations to gain extensive insight of the biological mechanisms underlying MetS and its related traits in the future.

## Data Availability

Publicly available datasets were analyzed in this study. This data can be found here: Summary statistics of association analyses are available from the corresponding author upon reasonable request.
