# Supplementary material for: Identification of shared genetic risks underlying metabolic syndrome and its related traits in the Korean population
Source: Front Genet. 2024 Jul 10;15:1417262. doi: 10.3389/fgene.2024.1417262 (PMC11266026; doi:10.3389/fgene.2024.1417262)
Supplement: Supplementary file 1 [file DataSheet1.PDF]

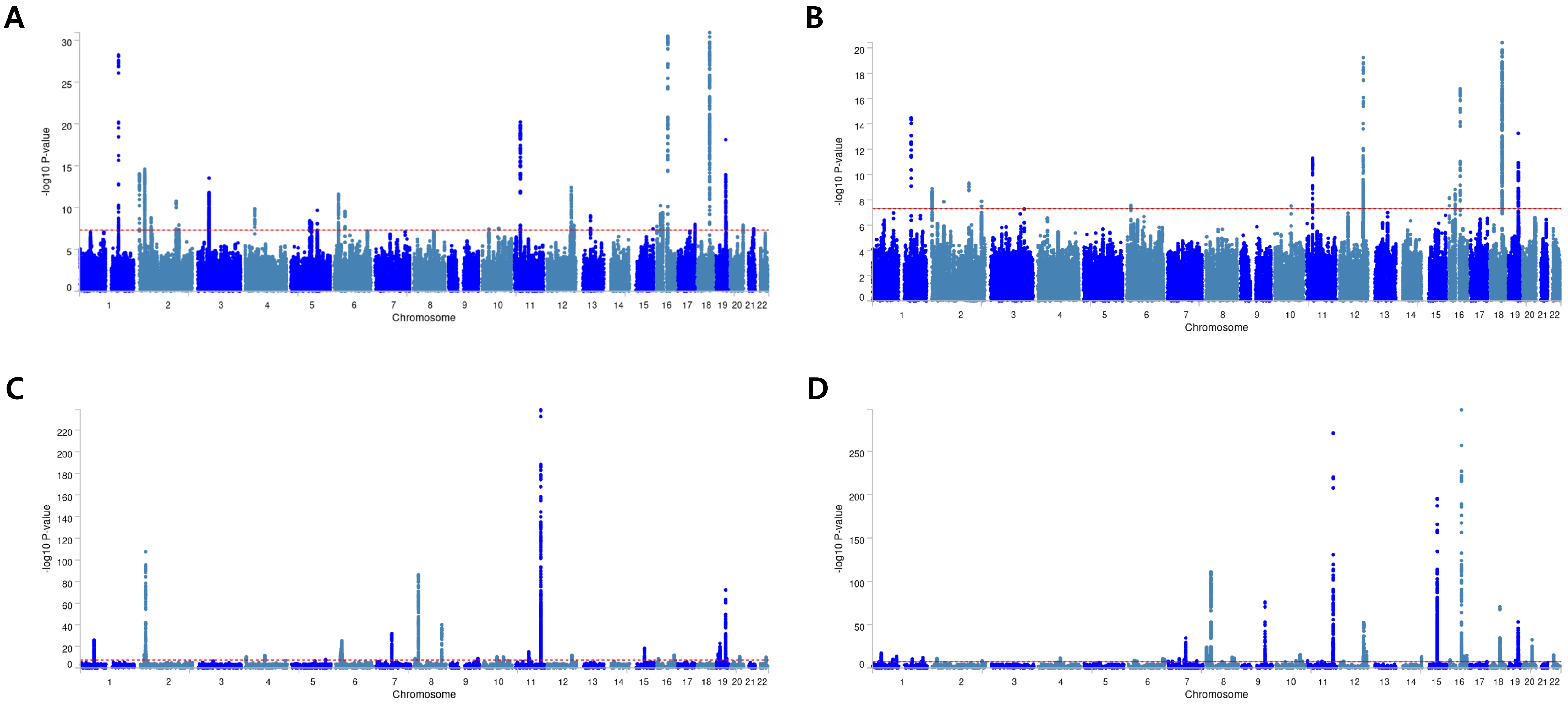
A
B
C
D
-log10 P-value
Chromosome

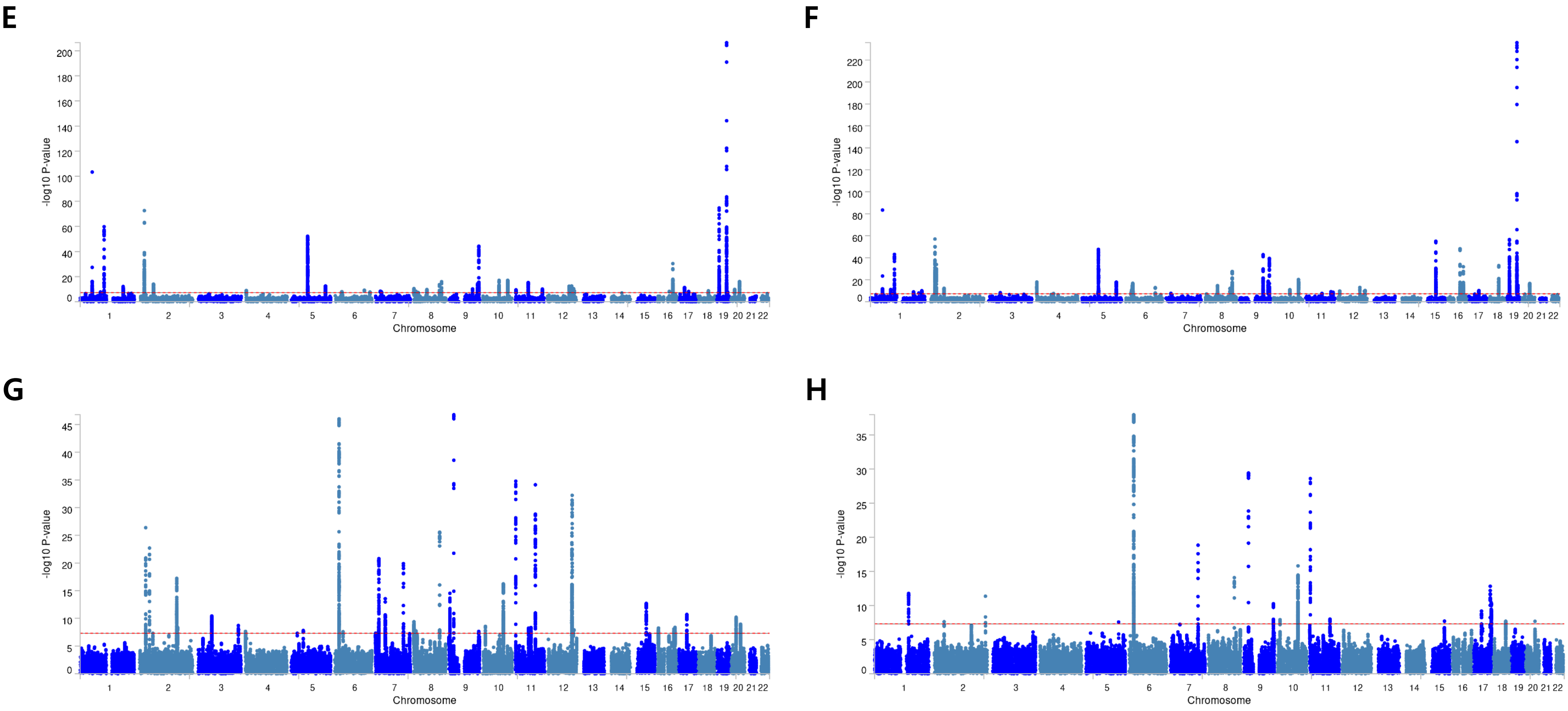

E
F
G
H
-log10 P-value
Chromosome

I

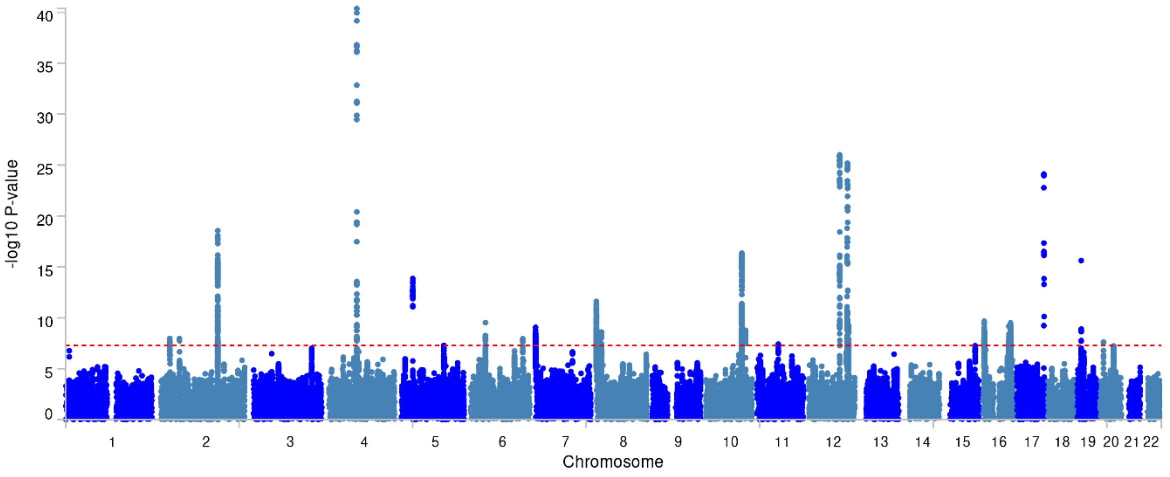

J

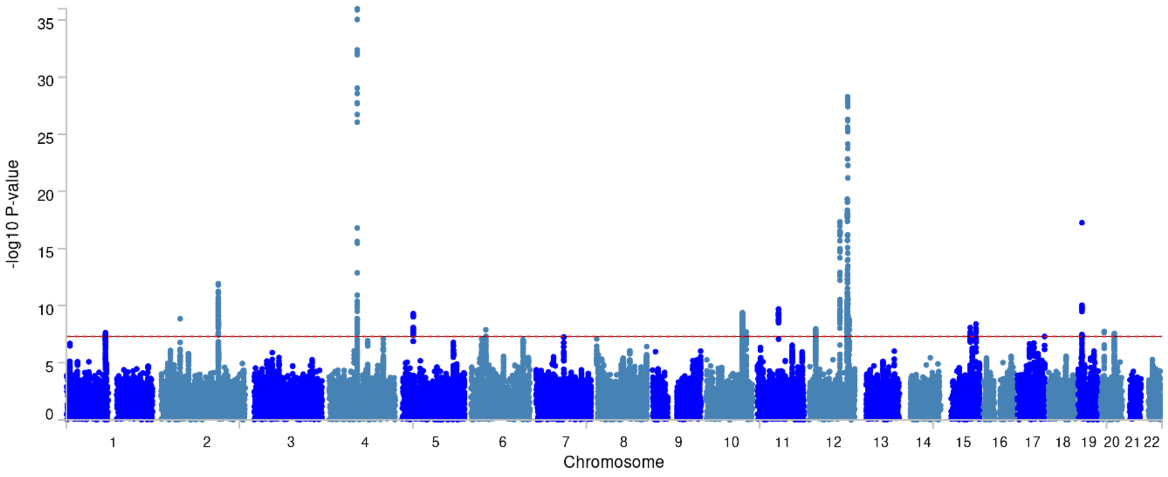

Supplementary Figure 1. Manhattan plots of GWA analyses of BMI (A), WC (B), TG (C), HDLC (D), LDLC (E), TCHL (F), FPG (G), HbA1C (H), SBP (I), and DBP (J).

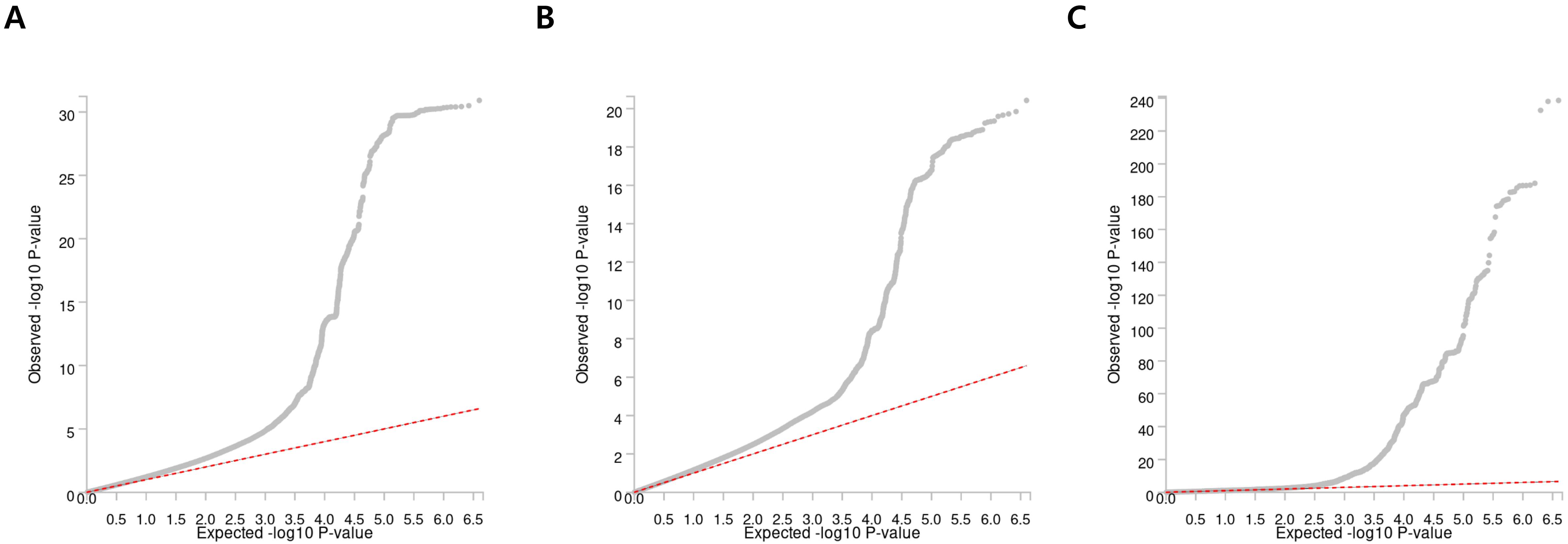
A
B
C
Observed -log10 P-value
Expected -log10 P-value
0.0
0.5
1.0
1.5
2.0
2.5
3.0
3.5
4.0
4.5
5.0
5.5
6.0
6.5
0
5
10
15
20
25
30
2
4
6
8
12
14
16
18
40
60
80
100
120
140
160
180
200
220
240

D

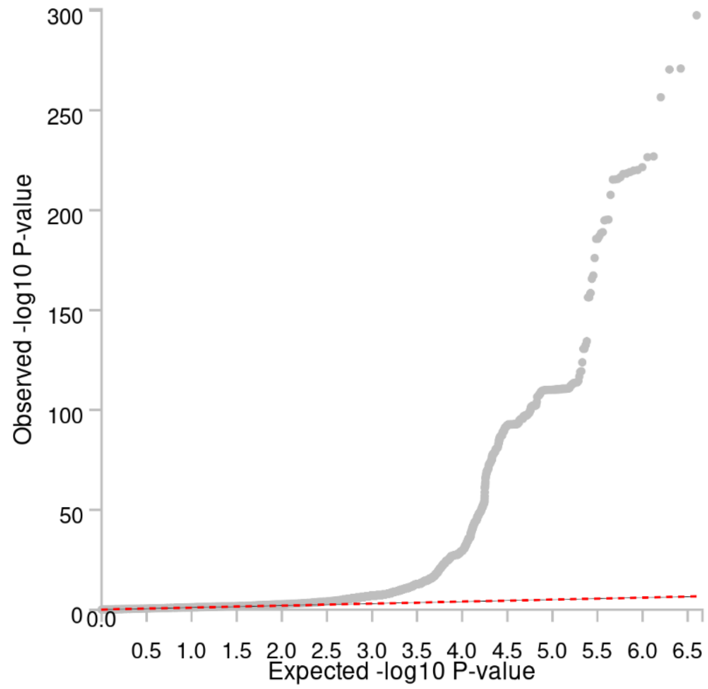
Observed -log10 P-value
300
250
200
150
100
50
0
0.0
0.5 1.0 1.5 2.0 2.5 3.0 3.5 4.0 4.5 5.0 5.5 6.0 6.5
Expected -log10 P-value

E

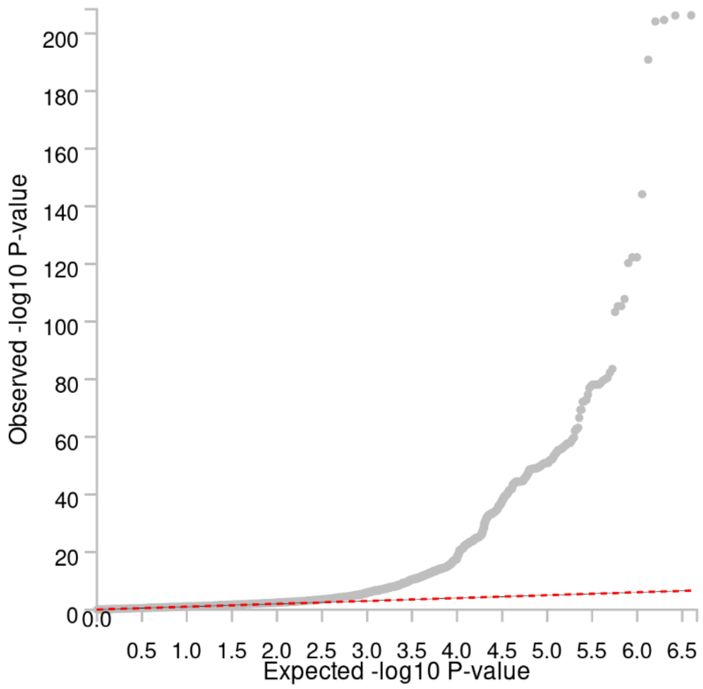
Observed -log10 P-value
200
180
160
140
120
100
80
60
40
20
0
0.0
0.5 1.0 1.5 2.0 2.5 3.0 3.5 4.0 4.5 5.0 5.5 6.0 6.5
Expected -log10 P-value

F

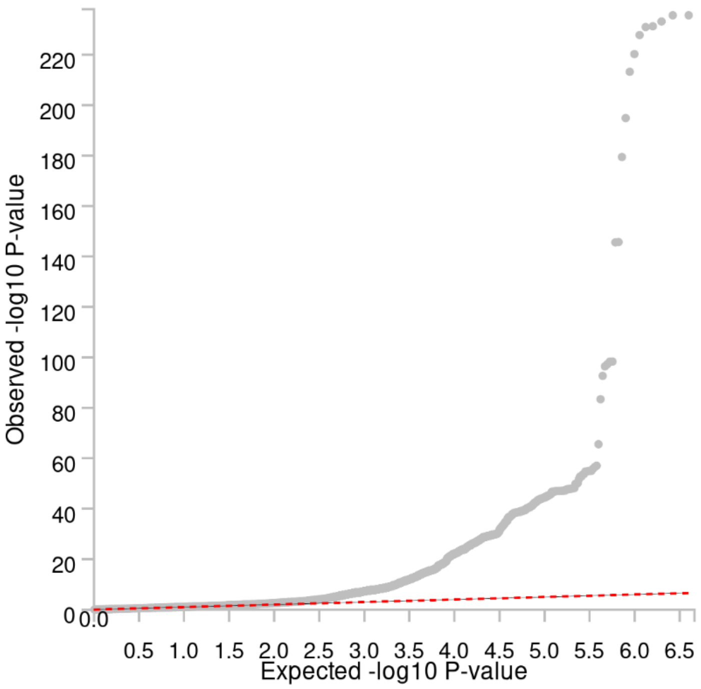
Observed -log10 P-value
220
200
180
160
140
120
100
80
60
40
20
0
0.0
0.5 1.0 1.5 2.0 2.5 3.0 3.5 4.0 4.5 5.0 5.5 6.0 6.5
Expected -log10 P-value

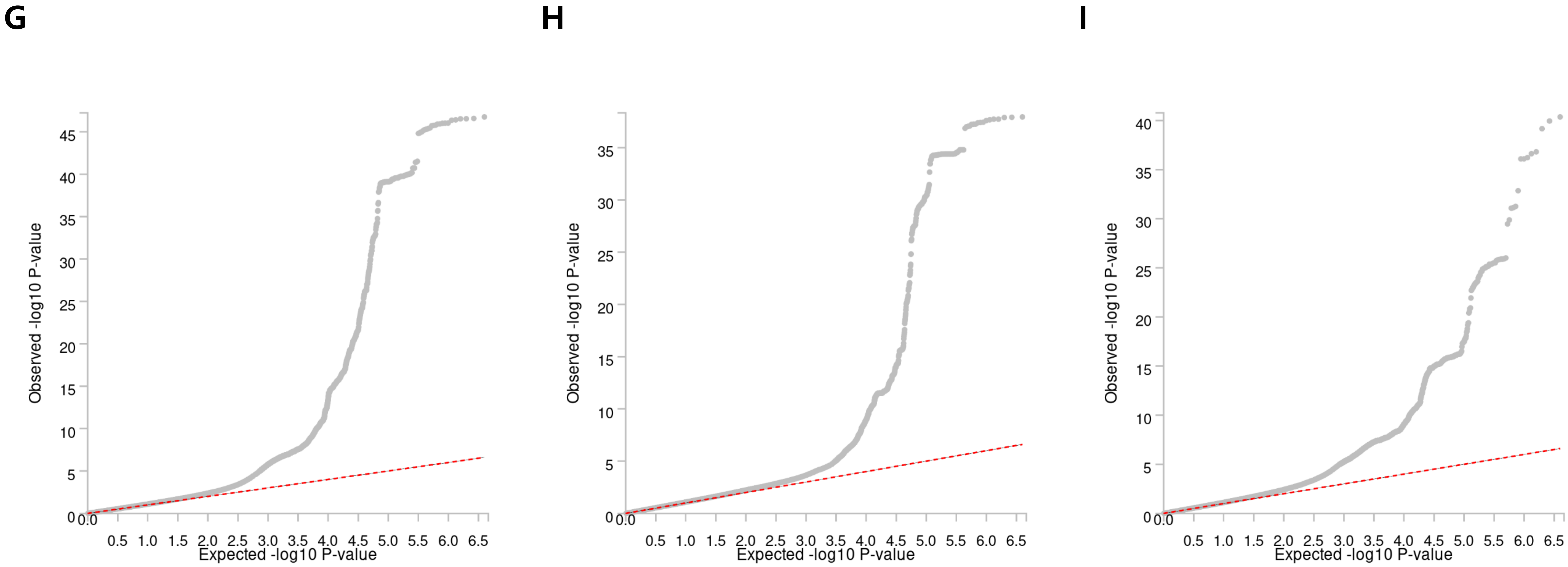
G
H
I
Observed -log10 P-value
Expected -log10 P-value
0.0
0.5
1.0
1.5
2.0
2.5
3.0
3.5
4.0
4.5
5.0
5.5
6.0
6.5
0
5
10
15
20
25
30
35
40
45

J

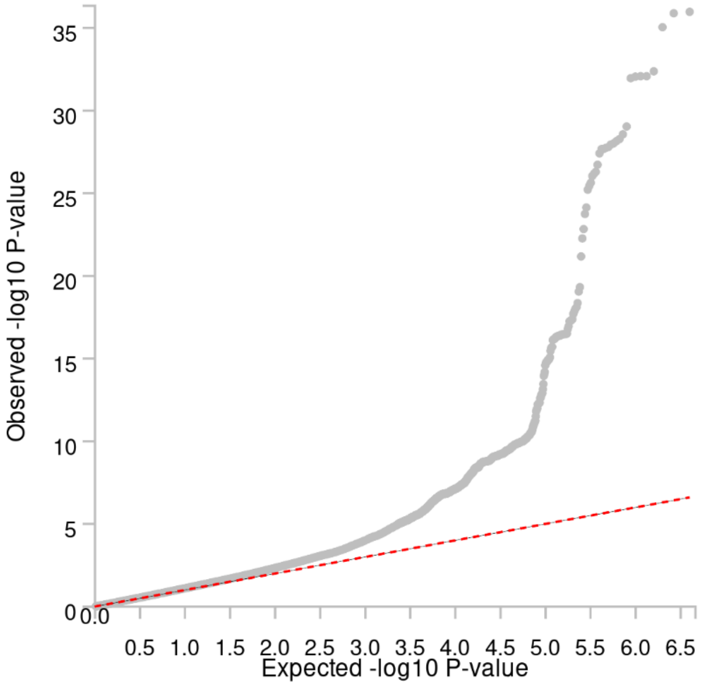

Supplementary Figure 2. Quantile-quantile plots of GWA analyses of BMI (A), WC (B), TG (C), HDLC (D), LDLC (E), TCHL (F), FPG (G), HbA1C (H), SBP (I), and DBP (J).

Supplementary Table 1. Clinical statistics of subjects with MetS-related diseases in the KBA dataset.

Trait	Variable	Case	Control
Obesity	N	17,023	39,429
	Age (year)	53.24	52.62
	BMI (kg/m2)	27.07	22.28
	WC (cm)	87.81	76.95
	TG (mg/dL)	151.60	114.34
	HDLC (mg/dL)	48.50	54.57
	LDLC (mg/dL)	122.25	118.19
	TCHL (mg/dL)	203.07	196.55
	FPG (mg/dL)	93.11	88.64
	HbA1C (%)	3.03	2.93
	SBP (mmHg)	125.34	118.69
	DBP (mmHg)	78.86	74.21
T2D	N	3,778	33,416
	Age (year)	56.51	52.92
	BMI (kg/m2)	24.86	23.71
	WC (cm)	85.10	80.05
	TG (mg/dL)	169.82	126.62
	HDLC (mg/dL)	47.36	52.88
	LDLC (mg/dL)	111.19	119.56
	TCHL (mg/dL)	196.08	198.84
	FPG (mg/dL)	133.75	90.36
	HbA1C (%)	4.43	4.72
	SBP (mmHg)	125.18	120.69
	DBP (mmHg)	77.36	75.61
Dyslipidemia	N	19,951	37,582
	Age (year)	54.27	52.19
	BMI (kg/m2)	24.55	23.30
	WC (cm)	83.05	78.76
	TG (mg/dL)	183.10	95.84
	HDLC (mg/dL)	46.16	56.25
	LDLC (mg/dL)	129.12	114.29
	TCHL (mg/dL)	215.08	189.71
	FPG (mg/dL)	92.77	88.60
	HbA1C (%)	3.07	2.93
	SBP (mmHg)	123.20	119.40
	DBP (mmHg)	77.48	74.61
Hypertension	N	17,429	48,372
	Age (year)	57.18	52.40
	BMI (kg/m2)	25.04	23.52
	WC (cm)	84.36	79.62
	TG (mg/dL)	148.18	120.79
	HDLC (mg/dL)	50.13	53.12
	LDLC (mg/dL)	118.69	119.17
	TCHL (mg/dL)	200.18	197.53
	FPG (mg/dL)	94.09	89.32
	HbA1C (%)	2.85	2.98
	SBP (mmHg)	137.04	116.84
	DBP (mmHg)	85.35	73.12

N, number of subjects; BMI, body mass index; WC, waist circumference; TG, triglyceride; HDLC, high-density lipoprotein cholesterol; LDLC, low-density lipoprotein cholesterol; TCHL, total cholesterol; FPG, fasting plasma glucose; HbA1C, hemoglobin A1c; SBP, systolic blood pressure; DBP, diastolic blood pressure.

Supplementary Table 2. Genetic variants showing evidence of an association with MetS.

CHR	Variant	BP(GRCh37)	Non-effect allele	Effect allele	MAF	OR	SE	Nearest Gene	Functional Consequence	*p* -value
11	11:116662579_C/T	116662579	T	C	0.288	1.401	0.016	*APOA5*	5'UTR	$4.27x10^{-96}$
16	16:56987369_T/C	56987369	T	C	0.183	0.828	0.021	*CETP*	intergenic	$2.20x10^{-19}$
8	8:19899046_C/T	19899046	C	T	0.340	0.867	0.016	*LPL*	intergenic	$5.81x10^{-18}$
19	19:45411941_T/C	45411941	T	C	0.086	1.154	0.025	*APOE*	exonic	$1.09x10^{-9}$

Information for chromosomal position of SNP is based on NCBI genome build 37/hg19. Abbreviations are as follows: MetS, metabolic syndrome; BP, base-pair (Physical position); MAF, minor allele frequency; OR, Odds ratio; SE, standard error.

Supplementary Table 3. Genetic variants showing evidence of an association with BMI.

CHR	Variant	BP(GRCh37)	Non-effect allele	Effect allele	MAF	BETA	SE	Nearest Gene	Functional Consequence	*p* -value
18	18:57852587_T/C	57852587	T	C	0.205	0.201	0.017	*RP11-795H16.2*	intergenic	$1.22x10^{-31}$
16	16:53809123_C/T	53809123	C	T	0.166	0.268	0.023	*FTO*	intronic	$3.18x10^{-31}$
1	1:177894591_T/G	177894591	T	G	0.177	0.188	0.017	*SEC16B*	intergenic	$5.85x10^{-29}$
11	11:27679916_C/T	27679916	C	T	0.488	-0.144	0.015	*BDNF-AS:BDNF*	exonic	$6.09x10^{-21}$
19	19:46175046_A/T	46175046	T	A	0.468	0.137	0.015	*GIPR*	intronic	$7.71x10^{-19}$
2	2:25158008_T/C	25158008	T	C	0.478	0.121	0.015	*DNAJC27*	intergenic	$2.68x10^{-15}$
2	2:630070_T/C	630070	C	T	0.092	-0.203	0.026	*TMEM18*	intergenic	$1.01x10^{-14}$
3	3:52866289_C/T	52866289	C	T	0.217	0.128	0.017	*RP5-966M1.6*	intronic	$3.00x10^{-14}$
12	12:112468206_C/T	112468206	C	T	0.172	-0.153	0.021	*NAA25*	intronic	$4.01x10^{-13}$
6	6:20680678_T/A	20680678	T	A	0.387	-0.107	0.015	*CDKAL1*	intronic	$2.51x10^{-12}$
2	2:169133796_A/G	169133796	A	G	0.330	0.112	0.017	*STK39*	intergenic	$1.70x10^{-11}$
16	16:20257273_A/G	20257273	A	G	0.191	-0.126	0.019	*SNRPEP3*	intergenic	$5.88x10^{-11}$
4	4:45182527_A/G	45182527	A	G	0.303	0.109	0.017	*RP11-362I1.1*	intergenic	$1.39x10^{-10}$
5	5:122699812_T/G	122699812	T	G	0.266	-0.109	0.017	*CEP120*	intronic	$2.17x10^{-10}$
6	6:50798526_C/T	50798526	C	T	0.234	0.104	0.016	*TFAP2B*	intronic	$2.87x10^{-10}$
16	16:30096468_T/TGGG	30096468	T	TGGG	0.367	0.102	0.016	*PPP4C*	3'UTR	$4.15x10^{-10}$
13	13:54082589_CAG/C	54082589	CAG	C	0.282	0.103	0.017	*AL450423.1*	intergenic	$9.85x10^{-10}$
2	2:54142030_T/C	54142030	T	C	0.012	-0.440	0.073	*PSME4*	intronic	$1.70x10^{-9}$
5	5:87970352_G/C	87970352	G	C	0.474	0.090	0.015	*LINC00461*	ncRNA_intronic	$3.69x10^{-9}$
19	19:47596073_C/CAATA	47596073	C	CAATA	0.454	0.090	0.015	*ZC3H4*	intronic	$4.99x10^{-9}$
5	5:95850250_A/C	95850250	A	C	0.465	0.090	0.015	*CTD-2337A12.1*	ncRNA_intronic	$6.00x10^{-9}$
17	17:79049527_G/A	79049527	G	A	0.320	0.090	0.016	*BAIAP2*	intronic	$1.07x10^{-8}$
2	2:181514955_A/C	181514955	C	A	0.430	-0.091	0.016	*AC009478.1*	ncRNA_intronic	$1.23x10^{-8}$
20	20:60585687_T/C	60585687	C	T	0.344	0.094	0.017	*TAF4*	intronic	$1.27x10^{-8}$
12	12:124576411_A/G	124576411	G	A	0.331	0.094	0.016	*FAM101A*	intronic	$1.32x10^{-8}$
16	16:4022694_C/T	4022694	C	T	0.313	0.089	0.016	*ADCY9*	intronic	$1.80x10^{-8}$
10	10:77437044_C/T	77437044	T	C	0.416	-0.084	0.015	*C10orf11*	intergenic	$3.04x10^{-8}$
21	21:40300688_C/T	40300688	T	C	0.320	0.100	0.018	*AF064858.6*	ncRNA_intronic	$3.52x10^{-8}$
15	15:98107153_G/A	98107153	G	A	0.229	0.102	0.019	*RP11-461F11.3*	intergenic	$3.65x10^{-8}$
10	10:31611680_CTT/CT	31611680	CTT	CT	0.195	-0.107	0.019	*ZEB1*	intronic	$3.89x10^{-8}$

Information for chromosomal position of SNP is based on NCBI genome build 37/hg19. Abbreviations are as follows: BMI, body mass index; BP, base-pair (Physical position); MAF, minor allele frequency; SE, standard error.

Supplementary Table 4. Genetic variants showing evidence of an association with WC.

CHR	Variant	BP(GRCh37)	Non-effect allele	Effect allele	MAF	BETA	SE	Nearest Gene	Functional Consequence	*p* -value
18	18:57829135_T/C	57829135	T	C	0.183	0.461	0.049	*RP11-795H16.3*	downstream	$3.76x10^{-21}$
12	12:112468206_C/T	112468206	C	T	0.172	-0.526	0.057	*NAA25*	intronic	$5.75x10^{-20}$
16	16:53803574_T/A	53803574	T	A	0.166	0.538	0.063	*FTO*	intronic	$1.66x10^{-17}$
1	1:177894591_T/G	177894591	T	G	0.177	0.363	0.046	*SEC16B*	intergenic	$3.34x10^{-15}$
19	19:46175046_A/T	46175046	T	A	0.468	0.318	0.042	*GIPR*	intronic	$5.63x10^{-14}$
11	11:27658138_G/GA	27658138	G	GA	0.414	-0.291	0.042	*BDNF-AS*	ncRNA_intronic	$5.25x10^{-12}$
2	2:169129145_G/T	169129145	G	T	0.329	0.283	0.045	*STK39*	intergenic	$4.79x10^{-10}$
2	2:650143_T/C	650143	T	C	0.087	-0.437	0.072	*TMEM18*	intergenic	$1.34x10^{-9}$
16	16:30096468_T/TGGG	30096468	T	TGGG	0.367	0.270	0.045	*PPP4C*	3'UTR	$1.52x10^{-9}$
16	16:4013467_C/T	4013467	C	T	0.301	0.251	0.043	*ADCY9*	3'UTR	$6.96x10^{-9}$
2	2:227231563_G/C	227231563	C	G	0.302	0.250	0.044	*AC068138.1*	intergenic	$1.32x10^{-8}$
2	2:54256536_C/A	54256536	A	C	0.479	0.238	0.042	*ACYP2*	intronic	$1.44x10^{-8}$
6	6:20686996_C/A	20686996	C	A	0.387	-0.231	0.042	*CDKAL1*	intronic	$2.76x10^{-8}$
10	10:77437044_C/T	77437044	T	C	0.416	-0.231	0.042	*C10orf11*	intergenic	$2.99x10^{-8}$

Information for chromosomal position of SNP is based on NCBI genome build 37/hg19. Abbreviations are as follows: WC, waist circumference; BP, base-pair (Physical position); MAF, minor allele frequency; SE, standard error.

Supplementary Table 5. Genetic variants showing evidence of an association with TG.

CHR	Variant	BP(GRCh37)	Non-effect allele	Effect allele	MAF	BETA	SE	Nearest Gene	Functional Consequence	*p*-value
11	11:116596894_C/T	116596894	C	T	0.028	33.170	1.000	*BUD13*	intergenic	2.36×10^{-239}
2	2:27730940_T/C	27730940	T	C	0.481	-10.200	0.461	*GCKR*	exonic	3.79×10^{-108}
8	8:19823674_C/T	19823674	C	T	0.120	-13.660	0.691	*LPL*	3'UTR	7.83×10^{-87}
19	19:45416741_C/T	45416741	C	T	0.202	11.120	0.616	*APOC1*	upstream	8.32×10^{-73}
8	8:126507389_C/A	126507389	A	C	0.267	6.774	0.506	*RP11-136O12.2*	ncRNA_intronic	9.48×10^{-41}
7	7:73034030_A/G	73034030	A	G	0.116	-8.894	0.751	*MLXIPL*	intronic	2.55×10^{-32}
1	1:63153199_TCAGAA/T	63153199	TCAGAA	T	0.177	-6.402	0.603	*DOCK7*	intronic	2.59×10^{-26}
19	19:19379549_C/T	19379549	C	T	0.086	-8.760	0.876	*C138430.4:TM6SF*	exonic	1.54×10^{-23}
15	15:58723675_C/T	58723675	C	T	0.400	4.131	0.464	*ALDH1A2:LIPC*	intronic	5.66×10^{-19}
6	6:29602522_C/A	29602522	C	A	0.019	8.238	0.991	*GABBR1*	upstream	9.51×10^{-17}
11	11:61624181_T/C	61624181	C	T	0.417	3.861	0.483	*FADS2*	intronic	1.35×10^{-15}
6	6:33619442_G/A	33619442	A	G	0.027	8.014	1.034	*ITPR3*	intronic	9.51×10^{-15}
19	19:11347493_T/C	11347493	T	C	0.240	-3.778	0.509	*DOCK6*	exonic	1.11×10^{-13}
2	2:21252534_G/A	21252534	G	A	0.128	-5.087	0.704	*APOB*	exonic	5.18×10^{-13}
16	16:81545764_G/T	81545764	G	T	0.358	-3.347	0.472	*CMIP*	intronic	1.39×10^{-12}
12	12:112930475_T/C	112930475	T	C	0.180	-4.398	0.623	*PTPN11*	intronic	1.72×10^{-12}
4	4:88076626_A/G	88076626	A	G	0.346	3.408	0.486	*KLHL8*	intergenic	2.36×10^{-12}
20	20:44557215_A/AAGAG	44557215	A	AAGAG	0.115	5.687	0.861	*PCIF1*	intergenic	3.93×10^{-11}
10	10:65323997_G/T	65323997	G	T	0.274	3.112	0.473	*REEP3*	intronic	4.89×10^{-11}
4	4:3447156_C/T	3447156	C	T	0.447	-2.997	0.460	*HGFAC*	intronic	7.19×10^{-11}
10	10:94839724_G/T	94839724	T	G	0.207	3.339	0.515	*CYP26A1*	intergenic	9.38×10^{-11}
22	22:44343626_A/C	44343626	A	C	0.365	-2.998	0.466	*PNPLA3*	intronic	1.25×10^{-10}
11	11:65276231_CT/C	65276231	CT	C	0.254	3.247	0.535	*MALAT1*	intergenic	1.28×10^{-9}
19	19:50043418_C/CTG	50043418	C	CTG	0.021	8.410	1.391	*RCN3*	intronic	1.50×10^{-9}
8	8:3971565_C/T	3971565	C	T	0.005	3.265	0.544	*CSMD1*	intronic	1.92×10^{-9}
9	9:130768150_A/ATTAAT	130768150	A	ATTAAT	0.135	3.957	0.660	*FAM102A*	intergenic	2.00×10^{-9}
16	16:9736651_G/A	9736651	G	A	0.001	3.093	0.518	*RP11-297M9.1*	intergenic	2.36×10^{-9}
8	8:18273300_G/C	18273300	G	C	0.485	-2.864	0.480	*NAT2*	intergenic	2.51×10^{-9}
5	5:156356294_T/C	156356294	T	C	0.053	-4.700	0.830	*TIMD4*	intronic	1.49×10^{-8}

Information for chromosomal position of SNP is based on NCBI genome build 37/hg19. Abbreviations are as follows: TG, triglyceride; BP, base-pair (Physical position); MAF, minor allele frequency; SE, standard error.

Supplementary Table 6. Genetic variants showing evidence of an association with HDLC.

CHR	Variant	BP(GRCh37)	Non-effect allele	Effect allele	MAF	BETA	SE	Nearest Gene	Functional Consequence	*p* -value
16	16:57001274_AC/A	57001274	AC	A	0.171	3.282	0.088	*CETP*	intronic	2.23×10^{-298}
11	11:116662579_C/T	116662579	T	C	0.288	-2.509	0.071	*APOA5*	5'UTR	1.04×10^{-271}
15	15:58723426_A/G	58723426	A	G	0.419	1.972	0.066	*ALDH1A2:LIPC*	intronic	4.63×10^{-196}
8	8:19847690_C/A	19847690	C	A	0.119	2.219	0.099	*LPL*	intergenic	1.71×10^{-111}
9	9:107665739_G/A	107665739	G	A	0.240	-1.404	0.076	*ABCA1*	intronic	1.06×10^{-76}
18	18:47120600_G/A	47120600	G	A	0.426	1.171	0.066	*LIPG*	intergenic	2.90×10^{-71}
19	19:45411941_T/C	45411941	T	C	0.086	-1.722	0.112	*APOE*	exonic	9.09×10^{-54}
12	12:112119171_T/C	112119171	T	C	0.173	-1.387	0.091	*BRAP*	intronic	6.55×10^{-53}
7	7:80286003_C/T	80286003	C	T	0.010	1.605	0.129	*CD36*	exonic	2.71×10^{-35}
20	20:44557215_A/AAGAG	44557215	A	AAGAG	0.115	-1.476	0.123	*PCIF1*	intergenic	3.62×10^{-33}
8	8:241362_T/A	241362	T	A	0.001	-1.671	0.163	*RP11-63E5.6*	intergenic	1.40×10^{-24}
12	12:125338529_C/T	125338529	T	C	0.382	-0.603	0.067	*SCARB1*	intronic	1.83×10^{-19}
1	1:40036847_G/C	40036847	G	C	0.101	-0.928	0.108	*PABPC4:RP11-69E11.8*	ncRNA_intronic	8.56×10^{-18}
10	10:113916835_A/C	113916835	C	A	0.240	0.576	0.071	*GPAM*	3'UTR	4.46×10^{-16}
22	22:21977047_C/T	21977047	C	T	0.492	-0.532	0.066	*UBE2L3*	3'UTR	1.23×10^{-15}
8	8:4845784_A/G	4845784	A	G	0.001	-1.473	0.185	*CSMD1*	intronic	1.41×10^{-15}
16	16:81534790_T/C	81534790	C	T	0.417	-0.535	0.068	*CMIP*	intronic	3.39×10^{-15}
16	16:68092130_C/CT	68092130	C	CT	0.100	0.692	0.091	*DUS2*	intronic	2.99×10^{-14}
1	1:110470764_G/A	110470764	G	A	0.281	-0.527	0.070	*CSF1*	intronic	5.56×10^{-14}
14	14:105268228_G/A	105268228	G	A	0.306	-0.506	0.069	*ZBTB42*	exonic	1.59×10^{-13}
8	8:116625707_C/T	116625707	T	C	0.143	-0.581	0.080	*TRPS1*	intronic	5.02×10^{-13}
1	1:230294916_C/T	230294916	C	T	0.195	0.634	0.089	*GALNT2*	intronic	1.37×10^{-12}
4	4:100445238_G/A	100445238	G	A	0.239	0.533	0.077	*C4orf17*	intronic	4.12×10^{-12}
8	8:126500031_C/G	126500031	C	G	0.141	0.556	0.081	*RP11-136O12.2*	ncRNA_intronic	6.43×10^{-12}
2	2:21179378_T/C	21179378	C	T	0.152	-0.620	0.091	*RP11-116D2.1*	intergenic	1.23×10^{-11}
6	6:160989288_C/T	160989288	C	T	0.500	-0.440	0.066	*LPA*	intronic	2.19×10^{-11}
7	7:50270105_T/A	50270105	T	A	0.403	0.441	0.066	*AC020743.3*	intergenic	2.86×10^{-11}
1	1:182119516_C/T	182119516	C	T	0.254	-0.475	0.072	*GS1-122H1.2*	ncRNA_intronic	3.86×10^{-11}
6	6:168243241_A/C	168243241	C	A	0.195	-0.551	0.086	*MLLT4*	intronic	1.31×10^{-10}
1	1:93825638_T/A	93825638	A	T	0.362	0.432	0.067	*DR1*	intronic	1.33×10^{-10}
7	7:73004315_C/T	73004315	C	T	0.087	0.711	0.113	*MLXIPL*	intergenic	3.47×10^{-10}
12	12:123191205_GA/G	123191205	G	GA	0.488	-0.418	0.067	*HCAR1:RP11-324E6.6*	ncRNA_intronic	4.13×10^{-10}
16	16:5280287_G/C	5280287	G	C	0.407	-0.464	0.074	*RP11-420N3.2*	intergenic	4.16×10^{-10}
16	16:76099909_A/T	76099909	A	T	0.273	-0.354	0.059	*RP11-293N14.1*	intergenic	1.59×10^{-9}
19	19:33899065_G/A	33899065	G	A	0.429	0.395	0.066	*PEPD*	intronic	1.82×10^{-9}
10	10:94746240_G/A	94746240	A	G	0.316	-0.399	0.068	*EXOC6*	intronic	3.84×10^{-9}
11	11:270333_A/T	270333	T	A	0.206	-0.469	0.081	*NLRP6*	intergenic	7.92×10^{-9}
7	7:130432913_A/AT	130432913	A	AT	0.315	0.413	0.072	*KLF14*	intergenic	1.01×10^{-8}
2	2:227168672_C/T	227168672	T	C	0.072	0.731	0.128	*AC068138.1*	intergenic	1.02×10^{-8}
10	10:122937358_C/A	122937358	C	A	0.315	0.406	0.072	*LINC01153*	upstream	1.97×10^{-8}
12	12:20461604_A/T	20461604	A	T	0.116	0.487	0.087	*RP11-284H19.1*	intergenic	2.49×10^{-8}
6	6:43758873_G/A	43758873	A	G	0.259	0.430	0.078	*VEGFA*	intergenic	2.86×10^{-8}
7	7:17914600_C/T	17914600	T	C	0.498	0.364	0.066	*SNX13*	intronic	3.02×10^{-8}
11	11:65406393_C/T	65406393	C	T	0.254	-0.441	0.080	*SIPA1*	intronic	3.56×10^{-8}
7	7:84101_G/A	84101	G	A	0.001	0.301	0.055	*AC093627.8*	intergenic	4.21×10^{-8}

Information for chromosomal position of SNP is based on NCBI genome build 37/hg19. Abbreviations are as follows: HDLC, high density lipoprotein cholesterol; BP, base-pair (Physical position); MAF, minor allele frequency; SE, standard error.

Supplementary Table 7. Genetic variants showing evidence of an association with LDLC.

CHR	Variant	BP(GRCh37)	Non-effect allele	Effect allele	MAF	BETA	SE	Nearest Gene	Functional Consequence	*p* -value
19	19:45387034_A/C	45387034	A	C	0.105	-9.233	0.300	*PVRL2:CTB-129P6.4*	ncRNA_intronic	$4.35x10^{-207}$
1	1:55509585_C/T	55509585	C	T	0.013	-14.870	0.685	*PCSK9*	exonic	$4.71x10^{-104}$
19	19:11242307_G/C	11242307	C	G	0.267	-3.418	0.186	*LDLR:SPC24*	3'UTR	$2.09x10^{-75}$
2	2:21242731_G/A	21242731	G	A	0.009	14.960	0.825	*APOB*	exonic	$2.44x10^{-73}$
1	1:109817590_G/T	109817590	G	T	0.043	-5.880	0.358	*CELSR2*	3'UTR	$1.67x10^{-60}$
5	5:74644706_T/G	74644706	G	T	0.473	-2.576	0.168	*HMGCR*	intronic	$7.26x10^{-53}$
9	9:136139907_GAAACTGCC/G	136139907	GAAACTGCC	G	0.191	2.688	0.191	*ABO*	ncRNA_intronic	$6.05x10^{-45}$
16	16:72079657_C/T	72079657	C	T	0.240	-2.268	0.195	*TXNL4B*	intronic	$4.21x10^{-31}$
10	10:113980428_T/C	113980428	T	C	0.418	-1.485	0.173	*GPAM*	intergenic	$8.35x10^{-18}$
10	10:74692646_T/G	74692646	T	G	0.258	-1.683	0.196	*OIT3*	3'UTR	$8.86x10^{-18}$
20	20:39845070_C/T	39845070	T	C	0.289	-1.615	0.194	*ZHX3*	intronic	$9.37x10^{-17}$
8	8:126490972_A/T	126490972	T	A	0.448	1.410	0.170	*RP11-136O12.2*	ncRNA_intronic	$1.10x10^{-16}$
11	11:61588305_A/G	61588305	A	G	0.435	-1.496	0.185	*FADS2:FADS1*	intronic	$5.82x10^{-16}$
9	9:130760328_G/GT	130760328	G	GT	0.186	1.691	0.216	*FAM102A*	intergenic	$4.34x10^{-15}$
2	2:62827879_G/C	62827879	G	C	0.185	-1.640	0.212	*AC092155.4*	ncRNA_intronic	$9.83x10^{-15}$
8	8:116658583_G/T	116658583	G	T	0.285	-1.432	0.188	*TRPS1*	intronic	$2.32x10^{-14}$
12	12:112119171_T/C	112119171	T	C	0.173	1.699	0.233	*BRAP*	intronic	$2.97x10^{-13}$
5	5:156394518_C/T	156394518	T	C	0.274	-1.460	0.200	*TIMD4*	intergenic	$3.24x10^{-13}$
12	12:100893636_C/T	100893636	C	T	0.318	1.329	0.184	*NR1H4*	exonic	$4.65x10^{-13}$
1	1:196816180_AT/A	196816180	AT	A	0.353	1.262	0.176	*CFHR2*	intronic	$7.43x10^{-13}$
17	17:26716646_T/C	26716646	T	C	0.244	-1.501	0.217	*SARM1*	intronic	$5.07x10^{-12}$
8	8:219410_C/T	219410	C	T	0.001	2.828	0.427	*RP11-63E5.1*	intergenic	$3.51x10^{-11}$
12	12:121416650_A/C	121416650	A	C	0.389	1.118	0.169	*HNF1A-AS1:HNF1A*	exonic	$4.19x10^{-11}$
9	9:107661742_A/C	107661742	A	C	0.243	-1.268	0.194	*ABCA1*	intronic	$6.31x10^{-11}$
11	11:126233669_G/C	126233669	G	C	0.336	1.245	0.191	*ST3GAL4*	intronic	$7.67x10^{-11}$
20	20:17596587_G/A	17596587	G	A	0.076	-2.053	0.320	*RRBP1*	exonic	$1.40x10^{-10}$
8	8:59406490_C/T	59406490	C	T	0.245	-1.189	0.187	*CYP7A1*	intronic	$1.81x10^{-10}$
11	11:5684402_C/G	5684402	C	G	0.423	-1.075	0.171	*TRIM5*	downstream	$3.25x10^{-10}$
6	6:135402339_T/C	135402339	T	C	0.246	-1.113	0.180	*HBS1L*	intronic	$6.43x10^{-10}$
4	4:3442011_G/C	3442011	G	C	0.442	1.032	0.170	*RGS12*	downstream	$1.14x10^{-9}$
18	18:47120600_G/A	47120600	G	A	0.426	1.014	0.169	*LIPG*	intergenic	$1.95x10^{-9}$
12	12:124872587_T/C	124872587	T	C	0.359	1.081	0.181	*NCOR2*	intronic	$2.43x10^{-9}$
7	7:21614206_G/C	21614206	G	C	0.191	1.168	0.198	*DNAH11*	intronic	$3.87x10^{-9}$
8	8:9181611_G/A	9181611	A	G	0.012	-4.722	0.806	*RP11-115J16.1*	ncRNA_intronic	$4.76x10^{-9}$
16	16:55866378_T/C	55866378	C	T	0.494	-0.990	0.170	*CES1*	intronic	$5.82x10^{-9}$
17	17:45548500_C/CTTTATTTATTTA	45548500	C	TTATTTATT	0.270	1.120	0.194	*MRPL45P2*	ncRNA_intronic	$7.49x10^{-9}$
19	19:46200670_C/T	46200670	C	T	0.076	-2.167	0.375	*QPCTL*	intronic	$7.50x10^{-9}$
7	7:26022414_A/C	26022414	C	A	0.320	1.054	0.184	*MIR148A*	intergenic	$1.06x10^{-8}$
1	1:93267953_A/ATT	93267953	ATT	A	0.050	-2.000	0.355	*EVI5*	intergenic	$1.75x10^{-8}$
19	19:50016759_C/T	50016759	C	T	0.188	-1.341	0.238	*FCGRT*	intronic	$1.80x10^{-8}$
6	6:160988675_C/T	160988675	T	C	0.089	1.492	0.265	*LPA*	intronic	$1.84x10^{-8}$
8	8:19860460_C/G	19860460	C	G	0.118	1.393	0.255	*LPL*	intergenic	$4.98x10^{-8}$

Information for chromosomal position of SNP is based on NCBI genome build 37/hg19. Abbreviations are as follows: LDLC, low density lipoprotein cholesterol; BP, base-pair (Physical position); MAF, minor allele frequency; SE, standard error.

Supplementary Table 8. Genetic variants showing evidence of an association with TCHL.

CHR	Variant	BP(GRCh37)	Non-effect allele	Effect allele	MAF	BETA	SE	Nearest Gene	Functional Consequence	*p* -value
19	19:45412079_C/T	45412079	C	T	0.100	-12.420	0.377	*APOEI3:II3:I44+I3:I42*	exonic	$2.15x10^{-236}$
1	1:55509585_C/T	55509585	C	T	0.013	-14.710	0.756	*PCSK9*	exonic	$3.72x10^{-84}$
2	2:21242731_G/A	21242731	G	A	0.009	14.610	0.911	*APOB*	exonic	$8.92x10^{-58}$
19	19:11242307_G/C	11242307	C	G	0.267	-3.270	0.205	*LDLR:SPC24*	3'UTR	$2.83x10^{-57}$
15	15:58723426_A/G	58723426	A	G	0.419	2.951	0.187	*ALDH1A2:LIPC*	intronic	$9.46x10^{-56}$
16	16:56990716_C/A	56990716	C	A	0.175	3.647	0.248	*AC012181.1*	upstream	$6.04x10^{-49}$
5	5:74648603_A/T	74648603	T	A	0.490	-2.714	0.186	*HMGCR*	intronic	$2.16x10^{-48}$
1	1:109817590_G/T	109817590	G	T	0.043	-5.484	0.395	*CELSR2*	3'UTR	$7.71x10^{-44}$
9	9:107664301_C/T	107664301	C	T	0.243	-2.965	0.214	*ABCA1*	intronic	$1.60x10^{-43}$
2	2:27730940_T/C	27730940	T	C	0.481	-2.299	0.187	*GCKR*	exonic	$1.33x10^{-34}$
18	18:47120600_G/A	47120600	G	A	0.426	2.249	0.186	*LIPG*	intergenic	$1.32x10^{-33}$
16	16:72079657_C/T	72079657	C	T	0.240	-2.562	0.216	*TXNL4B*	intronic	$1.45x10^{-32}$
8	8:126490972_A/T	126490972	T	A	0.448	2.063	0.187	*RP11-136O12.2*	ncRNA_intronic	$3.03x10^{-28}$
10	10:113980428_T/C	113980428	T	C	0.418	-1.786	0.190	*GPAM*	intergenic	$6.43x10^{-21}$
9	9:130760328_G/GT	130760328	G	GT	0.186	2.138	0.237	*FAM102A*	intergenic	$2.16x10^{-19}$
4	4:3434885_T/G	3434885	T	G	0.496	-1.637	0.186	*RGS12*	intronic	$1.31x10^{-18}$
5	5:156394518_C/T	156394518	T	C	0.274	-1.941	0.221	*TIMD4*	intergenic	$1.54x10^{-18}$
20	20:39887648_C/A	39887648	A	C	0.292	-1.802	0.213	*ZHX3*	intronic	$2.59x10^{-17}$
19	19:50016759_C/T	50016759	C	T	0.188	-2.102	0.262	*FCGRT*	intronic	$1.14x10^{-15}$
8	8:59406490_C/T	59406490	C	T	0.245	-1.624	0.205	*CYP7A1*	intronic	$2.69x10^{-15}$
12	12:100895150_C/CT	100895150	C	CT	0.317	1.501	0.202	*NR1H4*	intronic	$1.15x10^{-13}$
6	6:135402339_T/C	135402339	T	C	0.246	-1.469	0.199	*HBS1L*	intronic	$1.38x10^{-13}$
2	2:62827879_G/C	62827879	G	C	0.185	-1.684	0.234	*AC092155.4*	ncRNA_intronic	$5.59x10^{-13}$
8	8:116658583_G/T	116658583	G	T	0.285	-1.489	0.207	*TRPS1*	intronic	$6.10x10^{-13}$
1	1:92998556_C/CTT	92998556	CTT	C	0.064	-2.510	0.367	*EVI5*	intronic	$8.01x10^{-12}$
6	6:28282863_A/G	28282863	A	G	0.051	2.181	0.320	*RP5-874C20.6*	intergenic	$9.82x10^{-12}$
10	10:74692646_T/G	74692646	T	G	0.258	-1.467	0.216	*OIT3*	3'UTR	$1.07x10^{-11}$
12	12:124872587_T/C	124872587	T	C	0.359	1.309	0.200	*NCOR2*	intronic	$5.67x10^{-11}$
17	17:45567593_ATC/A	45567593	ATC	A	0.324	1.296	0.199	*MRPL45P2*	ncRNA_exonic	$7.08x10^{-11}$
1	1:234664365_T/C	234664365	T	C	0.296	1.384	0.214	*RP5-855F14.1*	ncRNA_exonic	$9.38x10^{-11}$
12	12:9065846_G/C	9065846	G	C	0.310	-1.309	0.206	*PHC1*	upstream	$1.97x10^{-10}$
6	6:33952531_G/A	33952531	G	A	0.008	3.290	0.521	*MIR1275*	intergenic	$2.76x10^{-10}$
12	12:121416650_A/C	121416650	A	C	0.389	1.176	0.187	*HNF1A-AS1:HNF1A*	exonic	$3.00x10^{-10}$
11	11:116663707_G/A	116663707	A	G	0.288	1.249	0.203	*APOA5*	upstream	$7.34x10^{-10}$
1	1:196816180_AT/A	196816180	AT	A	0.353	1.184	0.194	*CFHR2*	intronic	$1.04x10^{-9}$
11	11:126233669_G/C	126233669	G	C	0.336	1.258	0.211	*ST3GAL4*	intronic	$2.35x10^{-9}$
3	3:52600705_A/G	52600705	A	G	0.087	2.026	0.347	*SMIM4:PBRM1*	intronic	$5.48x10^{-9}$
1	1:220970028_A/G	220970028	G	A	0.232	-1.455	0.256	*45352*	exonic	$1.23x10^{-8}$
7	7:21614206_G/C	21614206	G	C	0.191	1.224	0.219	*DNAH11*	intronic	$2.16x10^{-8}$
19	19:46301611_C/T	46301611	C	T	0.078	-2.078	0.371	*RSPH6A*	intronic	$2.17x10^{-8}$
11	11:75449644_T/C	75449644	T	C	0.227	-1.221	0.220	*RN7SL786P*	intergenic	$2.93x10^{-8}$
4	4:79619199_C/A	79619199	C	A	0.103	-1.492	0.269	*LINC01094*	intergenic	$2.94x10^{-8}$

Information for chromosomal position of SNP is based on NCBI genome build 37/hg19. Abbreviations are as follows: TCHL, total cholesterol; BP, base-pair (Physical position); MAF, minor allele frequency; SE, standard error.

Supplementary Table 9. Genetic variants showing evidence of an association with FPG.

CHR	Variant	BP(GRCh37)	Non-effect allele	Effect allele	MAF	BETA	SE	Nearest Gene	Functional Consequence	*p* -value
9	9:22134253_G/A	22134253	G	A	0.437	-1.520	0.105	*CDKN2B-AS1*	intergenic	$1.75x10^{-47}$
6	6:20708220_GTATAT/G	20708220	GTATAT	G	0.382	1.494	0.104	*CDKAL1*	intronic	$9.51x10^{-47}$
11	11:2857233_G/A	2857233	G	A	0.369	-1.330	0.107	*KCNQ1*	intronic	$1.73x10^{-35}$
11	11:92708710_C/G	92708710	C	G	0.423	1.293	0.105	*MTNR1B*	intronic	$7.57x10^{-35}$
12	12:112645401_G/A	112645401	G	A	0.129	-1.783	0.149	*HECTD4*	intronic	$6.12x10^{-33}$
2	2:27730940_T/C	27730940	T	C	0.481	1.133	0.105	*GCKR*	exonic	$4.21x10^{-27}$
8	8:118185733_A/G	118185733	A	G	0.463	-1.121	0.106	*SLC30A8*	3'UTR	$2.73x10^{-26}$
2	2:45192080_G/C	45192080	G	C	0.445	1.071	0.107	*AC012354.6*	ncRNA_intronic	$2.00x10^{-23}$
7	7:15063430_T/A	15063430	T	A	0.316	-1.060	0.111	*AC006045.3*	intergenic	$1.62x10^{-21}$
7	7:127258384_C/T	127258384	C	T	0.121	1.890	0.203	*PAX4*	intergenic	$1.30x10^{-20}$
2	2:169771124_A/C	169771124	A	C	0.305	0.940	0.109	*SPC25*	intergenic	$5.96x10^{-18}$
10	10:94225734_T/C	94225734	T	C	0.006	2.252	0.269	*IDE*	intronic	$5.91x10^{-17}$
9	9:4283137_G/T	4283137	T	G	0.474	0.830	0.105	*GLIS3*	intronic	$2.96x10^{-15}$
7	7:44258964_G/T	44258964	G	T	0.475	-0.812	0.107	*CAMK2B*	3'UTR	$2.81x10^{-14}$
15	15:62395224_G/C	62395224	G	C	0.370	-0.766	0.104	*NPM1P47*	intergenic	$1.96x10^{-13}$
17	17:36099952_T/A	36099952	A	T	0.231	0.770	0.115	*HNF1B*	intronic	$1.93x10^{-11}$
3	3:63965093_C/A	63965093	A	C	0.478	0.689	0.104	*ATXN7*	intronic	$3.67x10^{-11}$
20	20:22562326_A/AG	22562326	A	AG	0.157	-0.991	0.152	*FOXA2*	3'UTR	$6.19x10^{-11}$
8	8:808633_G/A	808633	G	A	0.239	-0.585	0.094	*ERICH1-AS1*	ncRNA_intronic	$4.08x10^{-10}$
20	20:42823424_C/T	42823424	C	T	0.414	-0.647	0.106	*OSER1*	intergenic	$1.10x10^{-9}$
3	3:185503456_T/A	185503456	T	A	0.248	0.690	0.115	*IGF2BP2*	intronic	$1.97x10^{-9}$
10	10:12309139_C/G	12309139	C	G	0.461	0.622	0.105	*RN7SL232P*	intergenic	$2.73x10^{-9}$
7	7:13888699_G/C	13888699	G	C	0.403	0.630	0.107	*AC005019.3*	intergenic	$3.69x10^{-9}$
16	16:81250556_T/C	81250556	T	C	0.217	-0.527	0.090	*PKD1L2*	intronic	$4.20x10^{-9}$
2	2:173593150_G/C	173593150	C	G	0.477	-0.617	0.105	*RAPGEF4-AS1*	ncRNA_intronic	$4.49x10^{-9}$
11	11:72459564_C/CA	72459564	C	CA	0.088	-1.197	0.204	*ARAP1*	intronic	$4.52x10^{-9}$
16	16:5632426_G/C	5632426	G	C	0.109	-0.492	0.084	*RP11-420N3.2*	ncRNA_intronic	$5.54x10^{-9}$
11	11:61606642_A/G	61606642	A	G	0.431	-0.662	0.114	*FADS2*	intronic	$5.81x10^{-9}$
16	16:76004399_ATAT/A	76004399	ATAT	A	0.196	-0.484	0.084	*RNA5SP430*	intergenic	$8.31x10^{-9}$
12	12:118399491_A/T	118399491	A	T	0.429	0.601	0.105	*KSR2*	intronic	$1.20x10^{-8}$
12	12:121363506_A/G	121363506	A	G	0.020	1.800	0.316	*RPL12P33*	intergenic	$1.25x10^{-8}$
5	5:55808342_C/T	55808342	T	C	0.488	-0.590	0.104	*AC022431.2*	intronic	$1.53x10^{-8}$
8	8:10148928_GA/G	10148928	GA	G	0.134	-0.458	0.081	*MSRA*	intronic	$1.89x10^{-8}$
9	9:136155000_C/T	136155000	C	T	0.187	0.663	0.119	*ABO*	intergenic	$2.25x10^{-8}$
4	4:1309416_C/T	1309416	C	T	0.425	-0.598	0.107	*MAEA*	intronic	$2.30x10^{-8}$
6	6:39046655_C/T	39046655	C	T	0.230	-0.713	0.128	*GLP1R*	intronic	$2.57x10^{-8}$
7	7:77509_C/G	77509	C	G	0.072	-0.420	0.077	*AC093627.8*	ncRNA_intronic	$4.72x10^{-8}$
5	5:28292164_G/T	28292164	G	T	0.005	2.798	0.513	*CTD-2061E9.1*	intergenic	$4.91x10^{-8}$
2	2:60586707_C/G	60586707	G	C	0.349	-0.599	0.110	*AC007381.2*	ncRNA_exonic	$4.95x10^{-8}$

Information for chromosomal position of SNP is based on NCBI genome build 37/hg19. Abbreviations are as follows: FPG, fasting plasma glucose, base-pair (Physical position); MAF, minor allele frequency; SE, standard error.

Supplementary Table 10. Genetic variants showing evidence of an association with HbA1C.

CHR	Variant	BP(GRCh37)	Non-effect allele	Effect allele	MAF	BETA	SE	Nearest Gene	Functional Consequence	*p* -value
6	6:20688121_T/A	20688121	T	A	0.385	0.069	0.005	*CDKAL1*	intronic	$1.14x10^{-38}$
9	9:22132698_T/C	22132698	T	C	0.440	-0.061	0.005	*CDKN2B-AS1*	intergenic	$3.86x10^{-30}$
11	11:2858440_G/A	2858440	G	A	0.374	-0.062	0.005	*KCNQ1*	intronic	$2.55x10^{-29}$
7	7:127258384_C/T	127258384	C	T	0.121	0.094	0.010	*PAX4*	intergenic	$1.45x10^{-19}$
10	10:94466439_A/G	94466439	G	A	0.128	0.062	0.007	*Y_RNA*	intergenic	$1.57x10^{-16}$
8	8:118185733_A/G	118185733	A	G	0.463	-0.042	0.005	*SLC30A8*	3'UTR	$8.28x10^{-15}$
17	17:76124810_A/AG	76124810	A	AG	0.227	0.053	0.007	*TMC6*	5'UTR	$1.51x10^{-13}$
1	1:156298188_G/A	156298188	G	A	0.224	-0.045	0.006	*CCT3*	intronic	$1.70x10^{-12}$
2	2:234191103_T/A	234191103	T	A	0.024	0.109	0.016	*ATG16L1*	intronic	$4.34x10^{-12}$
17	17:80695406_G/A	80695406	G	A	0.313	-0.037	0.006	*FN3K*	intronic	$3.79x10^{-11}$
9	9:136155000_C/T	136155000	C	T	0.187	0.040	0.006	*ABO*	intergenic	$5.49x10^{-11}$
17	17:36099952_T/A	36099952	A	T	0.231	0.036	0.006	*HNF1B*	intronic	$6.48x10^{-10}$
11	11:92708710_C/G	92708710	C	G	0.423	0.031	0.005	*MTNR1B*	intronic	$9.79x10^{-9}$
10	10:12307894_C/T	12307894	C	T	0.460	0.030	0.005	*RN7SL232P*	intergenic	$1.19x10^{-8}$
15	15:77781926_A/G	77781926	A	G	0.383	0.030	0.005	*HMG20A*	intergenic	$1.98x10^{-8}$
18	18:57829135_T/C	57829135	T	C	0.183	0.035	0.006	*RP11-795H16.3*	downstream	$2.01x10^{-8}$
20	20:42822149_CA/C	42822149	CA	C	0.372	-0.031	0.006	*OSER1*	intergenic	$2.05x10^{-8}$
2	2:45184405_GT/G	45184405	GT	G	0.452	0.030	0.005	*AC012354.6*	ncRNA_intronic	$2.41x10^{-8}$
5	5:150205975_G/A	150205975	G	A	0.003	0.119	0.021	*IRGM*	intergenic	$2.60x10^{-8}$

Information for chromosomal position of SNP is based on NCBI genome build 37/hg19. Abbreviations are as follows: HbA1C, hemoglobin A1c, base-pair (Physical position); MAF, minor allele frequency; SE, standard error.

Supplementary Table 11. Genetic variants showing evidence of an association with SBP.

CHR	Variant	BP(GRCh37)	Non-effect allele	Effect allele	MAF	BETA	SE	Nearest Gene	Functional Consequence	*p* -value
4	4:81184341_A/T	81184341	A	T	0.360	1.103	0.082	*FGF5*	intergenic	$4.36x10^{-41}$
12	12:90069276_T/G	90069276	T	G	0.316	-0.864	0.081	*ATP2B1*	intronic	$9.94x10^{-27}$
12	12:112241766_G/A	112241766	G	A	0.174	-1.137	0.108	*ALDH2*	exonic	$6.45x10^{-26}$
17	17:78358945_G/A	78358945	G	A	0.002	3.871	0.376	*RNF213:CTD-2047H16.4*	exonic	$7.67x10^{-25}$
2	2:165008166_C/G	165008166	G	C	0.428	-0.707	0.079	*AC092684.1*	ncRNA_intronic	$2.78x10^{-19}$
10	10:104661245_C/T	104661245	C	T	0.289	-0.756	0.090	*C10orf32-ASMT:AS3MT*	3'UTR	$4.37x10^{-17}$
19	19:11526765_G/T	11526765	G	T	0.478	-0.641	0.078	*RGL3*	exonic	$2.41x10^{-16}$
5	5:32829929_T/C	32829929	C	T	0.341	-0.617	0.080	*NPR3:AC026703.1*	intergenic	$1.38x10^{-14}$
8	8:238353_G/T	238353	G	T	0.001	-1.708	0.244	*RP11-63E5.6*	intergenic	$2.41x10^{-12}$
16	16:5408060_G/A	5408060	G	A	0.271	-0.419	0.066	*RP11-420N3.2*	ncRNA_intronic	$2.17x10^{-10}$
6	6:43407144_A/AT	43407144	AT	A	0.412	0.529	0.084	*ABCC10*	intronic	$2.99x10^{-10}$
16	16:81148663_T/C	81148663	T	C	0.188	-0.408	0.065	*PKD1L2*	intronic	$3.14x10^{-10}$
12	12:116212862_T/A	116212862	T	A	0.372	-0.532	0.086	*RP11-110L15.1*	ncRNA_intronic	$5.80x10^{-10}$
16	16:76530941_G/C	76530941	G	C	0.001	0.405	0.066	*CNTNAP4*	intronic	$6.60x10^{-10}$
7	7:75339_C/T	75339	C	T	0.384	-0.423	0.069	*AC093627.8*	intergenic	$8.61x10^{-10}$
10	10:115721179_G/A	115721179	G	A	0.144	0.707	0.117	*NHLRC2*	intergenic	$1.67x10^{-9}$
8	8:15017046_C/T	15017046	C	T	0.404	-0.461	0.077	*SGCZ*	intronic	$2.47x10^{-9}$
2	2:26929282_T/G	26929282	T	G	0.237	-0.537	0.094	*KCNK3*	intronic	$1.06x10^{-8}$
2	2:54734484_G/A	54734484	G	A	0.022	1.186	0.207	*SPTBN1*	intronic	$1.09x10^{-8}$
6	6:151019061_GTT/G	151019061	G	GTT	0.079	-1.176	0.206	*PLEKHG1*	intronic	$1.15x10^{-8}$
8	8:10100055_T/A	10100055	A	T	0.007	-0.323	0.057	*MSRA*	intronic	$1.63x10^{-8}$
20	20:10965998_G/A	10965998	A	G	0.460	-0.437	0.078	*RP11-103J8.1*	ncRNA_intronic	$2.35x10^{-8}$
8	8:4911422_G/C	4911422	G	C	0.079	-0.865	0.155	*RP11-221H10.1*	intergenic	$2.60x10^{-8}$
11	11:61272309_C/T	61272309	C	T	0.431	-0.431	0.078	*MIR4488*	intergenic	$3.85x10^{-8}$

Information for chromosomal position of SNP is based on NCBI genome build 37/hg19. Abbreviations are as follows: SBP, systolic blood pressure; BP, base-pair (Physical position); MAF, minor allele frequency; SE, standard error.

Supplementary Table 12. Genetic variants showing evidence of an association with DBP.

CHR	Variant	BP(GRCh37)	Non-effect allele	Effect allele	MAF	BETA	SE	Nearest Gene	Functional Consequence	*p* -value
4	4:81184341_A/T	81184341	A	T	0.360	0.688	0.054	*FGF5*	intergenic	$1.07x10^{-36}$
12	12:112119171_T/C	112119171	T	C	0.173	-0.800	0.072	*BRAP*	intronic	$5.30x10^{-29}$
12	12:89978233_C/T	89978233	C	T	0.357	-0.459	0.053	*RP11-981P6.1*	ncRNA_intronic	$4.72x10^{-18}$
19	19:11526765_G/T	11526765	G	T	0.478	-0.447	0.052	*RGL3*	exonic	$5.54x10^{-18}$
2	2:165005726_T/C	165005726	T	C	0.407	-0.373	0.052	*AC092684.1*	ncRNA_intronic	$1.21x10^{-12}$
11	11:61272309_C/T	61272309	C	T	0.431	-0.331	0.052	*MIR4488*	intergenic	$1.98x10^{-10}$
10	10:104913653_G/A	104913653	G	A	0.265	-0.386	0.062	*NT5C2*	intronic	$4.18x10^{-10}$
5	5:32829929_T/C	32829929	C	T	0.341	-0.330	0.053	*NPR3:AC026703.1*	intergenic	$5.00x10^{-10}$
2	2:55045000_AC/A	55045000	AC	A	0.066	0.634	0.105	*EML6*	intronic	$1.40x10^{-9}$
12	12:116203759_A/C	116203759	C	A	0.380	-0.335	0.055	*RP11-110L15.1*	ncRNA_intronic	$1.56x10^{-9}$
15	15:91421283_T/C	91421283	T	C	0.086	0.514	0.087	*FURIN*	intronic	$4.06x10^{-9}$
15	15:75057745_CA/C	75057745	CA	C	0.186	-0.385	0.067	*CYP1A2*	intergenic	$8.22x10^{-9}$
12	12:20222678_C/T	20222678	T	C	0.495	-0.296	0.052	*RP11-664H17.1*	ncRNA_intronic	$1.06x10^{-8}$
6	6:43397110_T/G	43397110	G	T	0.441	0.314	0.055	*ABCC10*	intronic	$1.29x10^{-8}$
20	20:10965998_G/A	10965998	A	G	0.460	-0.292	0.052	*RP11-103J8.1*	ncRNA_intronic	$1.81x10^{-8}$
10	10:115725280_A/G	115725280	A	G	0.142	0.448	0.080	*NHLRC2*	intergenic	$2.00x10^{-8}$
1	1:113169667_T/G	113169667	T	G	0.440	0.290	0.052	*CAPZA1*	intronic	$2.32x10^{-8}$
20	20:40217726_T/C	40217726	T	C	0.189	-0.342	0.061	*CHD6*	intronic	$2.76x10^{-8}$

Information for chromosomal position of SNP is based on NCBI genome build 37/hg19. Abbreviations are as follows: DBP, diastolic blood pressure; BP, base-pair (Physical position); MAF, minor allele frequency; SE, standard error.

Supplementary Table 13. Genome-wide genetic correlations between MetS component diseases and related QTs.

Trait 1	Trait 2	r_g	r_g SE	P-value
Obesity	BMI	1.000	0.021	< 0.001
	WC	0.846	0.033	5.98×10^{-147}
	TG	0.111	0.071	1.18×10^{-1}
	HDLC	-0.120	0.067	7.38×10^{-2}
	LDLC	-0.014	0.072	8.44×10^{-1}
	TCHL	-0.004	0.066	9.53×10^{-1}
	FPG	0.187	0.078	1.60×10^{-2}
	HbA1C	0.205	0.085	1.55×10^{-2}
	SBP	0.190	0.080	1.80×10^{-2}
	DBP	0.086	0.069	2.09×10^{-1}
T2D	BMI	0.207	0.077	6.80×10^{-3}
	WC	0.269	0.083	1.20×10^{-3}
	TG	0.177	0.076	2.01×10^{-2}
	HDLC	-0.159	0.077	3.77×10^{-2}
	LDLC	-0.017	0.079	8.35×10^{-1}
	TCHL	-0.004	0.076	9.59×10^{-1}
	FPG	0.806	0.051	2.17×10^{-56}
	HbA1C	0.934	0.052	1.52×10^{-71}
	SBP	0.137	0.079	8.15×10^{-2}
	DBP	0.076	0.077	3.22×10^{-1}
Dyslipidemia	BMI	0.176	0.053	8.00×10^{-4}
	WC	0.238	0.060	7.02×10^{-5}
	TG	0.920	0.063	6.75×10^{-49}
	HDLC	-0.490	0.085	8.73×10^{-9}
	LDLC	0.514	0.100	2.71×10^{-7}
	TCHL	0.660	0.093	1.06×10^{-12}
	FPG	0.276	0.095	3.70×10^{-3}
	HbA1C	0.326	0.085	1.00×10^{-4}
	SBP	0.120	0.079	1.28×10^{-1}
	DBP	0.215	0.083	3.10×10^{-3}
Hypertension	BMI	0.264	0.062	2.24×10^{-5}
	WC	0.284	0.061	3.69×10^{-6}
	TG	0.252	0.077	$1.00 \text{X} 10^{-3}$
	HDLC	-0.227	0.105	3.03×10^{-2}
	LDLC	-0.041	0.084	6.28×10^{-1}
	TCHL	0.002	0.074	9.81×10^{-1}
	FPG	0.271	0.089	2.50×10^{-3}
	HbA1C	0.210	0.080	8.90×10^{-3}
	SBP	0.977	0.039	1.36×10^{-135}
	DBP	0.940	0.049	3.94×10^{-83}

Abbreviations are as follows: QT, quantitative trait; r_g, genetic correlation; SE, standard error; T2D, type 2 diabetes; BMI, body mass index; WC, waist circumference; TG, triglyceride; HDLC, high-density lipoprotein cholesterol; LDLC, low-density lipoprotein cholesterol; TCHL, total cholesterol; FPG, fasting plasma glucose; HbA1C, hemoglobin A1c; SBP, systolic blood pressure; DBP, diastolic blood pressure.
